# Dielectric metamaterials with effective self-duality and full-polarization omnidirectional brewster effect

**DOI:** 10.1038/s41377-024-01605-z

**Published:** 2024-09-20

**Authors:** Hao Luo, Jie Luo, Zhihui Zhang, Chao Wu, Quan Li, Wei Liu, Ruwen Peng, Mu Wang, Hongqiang Li, Yun Lai

**Affiliations:** 1 National Laboratory of Solid State Microstructures, School of Physics, and Collaborative Innovation Center of Advanced Microstructures, Nanjing University, Nanjing, 210093 China; 2 School of Physical Science and Technology & Jiangsu key Laboratory of Frontier Material Physics and Devices, Soochow University, Suzhou, 215006 China; 3 School of Physics Science and Engineering, Tongji University, Shanghai, 200092 China; 4 College of Electronic and Information Engineering, Tongji University, Shanghai, 200092 China; 5 College for Advanced Interdisciplinary Studies, National University of Defense Technology, Changsha, Hunan 410073 China

**Keywords:** Metamaterials, Sub-wavelength optics

## Abstract

Conventional dielectric solid materials, both natural and artificial, lack electromagnetic self-duality and thus require additional coatings to achieve impedance matching with free space. Here, we present a class of dielectric metamaterials that are effectively self-dual and vacuum-like, thereby exhibiting full-polarization omnidirectional impedance matching as an unusual Brewster effect extended across all incident angles and polarizations. With both birefringence and reflection eliminated regardless of wavefront and polarization, such anisotropic metamaterials could establish the electromagnetic equivalence with “stretched free space” in transformation optics, as substantiated through full-wave simulations and microwave experiments. Our findings open a practical pathway for realizing unprecedented polarization-independence and omnidirectional impedance-matching characteristics in pure dielectric solids.

## Introduction

The electromagnetic self-duality of free space, referring to the invariance of free-space Maxwell’s equations under the exchange of **E** → **H** and **H** → **-E**, plays an essential role across different scientific disciplines, such as physical singularities, gauge field theory, and string theory^1,2^. While in condensed matter like solids and liquids, this self-dual property is broken due to the imbalance between electric and magnetic responses of matter. The far-reaching consequences of this fundamental symmetry breaking include the classical Brewster effect, where polarization-dependent and angle-dependent non-reflection occurs on a dielectric surface^3^, and the classical birefringence^3^, where the refraction also becomes polarization-dependent, causing splitting beams. The polarization dependence is a signature of the absence of duality symmetry^4^.

For decades people have been trying to exploit artificial materials with balanced electric and magnetic responses to restore the duality symmetry^4–8^, but so far most studies were limited to theories. Recent studies suggest that equal electric and magnetic polarizabilities^9–18^ enable a plethora of novel phenomena including conservation of helicity^9^, directional scattering and Kerker effect^10,11^, scattering invariance^12,13^, Huygens’ metasurfaces^14–16^, etc. However, these realizations were mostly limited to particle or surface scatterings^17,18^. To date, self-dual solid materials have not yet been implemented in pure dielectric systems.

**Fig. 1:**
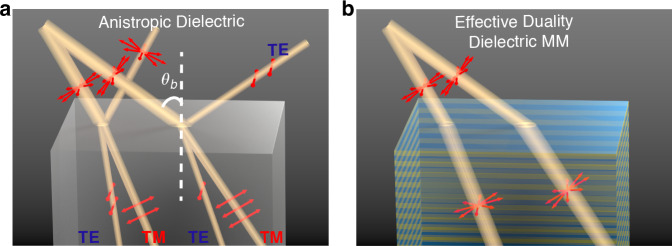
Dielectric MM exhibiting effective self-dual property and full-polarization omnidirectional Brewster effect. **a** The classical Brewster effect limited to a particular incident angle, i.e., Brewster angle ${\theta }_{{\rm{b}}}$ and the TM polarization, and the birefringence effect of beam splitting due to anisotropy. **b** Illustration of an anisotropic dielectric MM exhibiting full-polarization omnidirectional Brewster effect, as well as birefringence-free anisotropy as the consequence of effective duality symmetry realized in this dielectric MM

In this work, we introduce a class of dielectric metamaterials (MMs) with two distinct properties, (1) effective self-dual property and (2) full-polarization omnidirectional Brewster effect, as depicted in Fig. 1b. Over the past decades, the advent of dielectric MMs^19–26^ has opened a plethora of remarkable phenomena, including pure-dielectric impedance-matched transformation-optics devices^27–29^ and universal impedance matching layers^23^. Here, we present a systematic method to imbue dielectric MMs with effective self-duality, effectively mitigating birefringence and other polarization-dependent behaviors. Simultaneously, this MM achieves omnidirectional impedance-matching with free space across arbitrary incident angles and polarizations. Through numerical simulations and microwave experiments, we demonstrate that such a dielectric MM is akin to “stretched free space”^27^ in transformation optics^28–30^, exhibiting negligible reflection and birefringence. Our work thus unveils a route to remove the intrinsic features of polarization dependence and impedance mismatch with free space in dielectric solids, which could have unprecedented applications such as perfect solid radomes resembling free space.

## Results

**Fig. 2:**
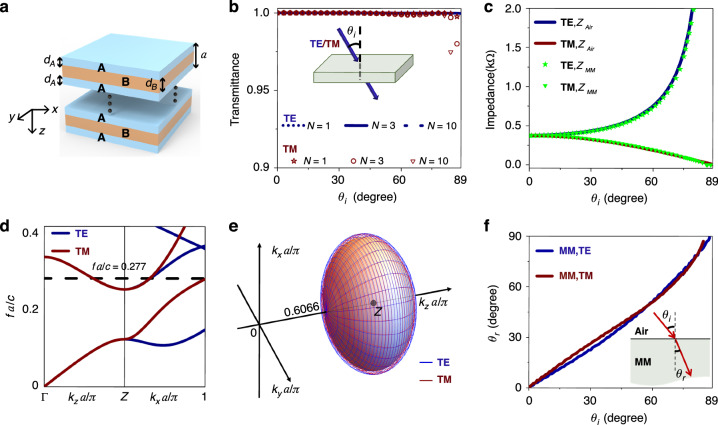
Design of effective self-dual MMs using dielectric multilayers. **a** Illustration of a dielectric MM consisting of ABA multilayers. **b** Transmittance through $N$ numbers of ABA units in air as a function of the incident angle ${\theta }_{i}$ for TE (blue) and TM (red) polarizations. **c** Wave impedances of free space (lines) and MM (dots) for TE and TM polarizations. **d** Band structures of the MM for TE (blue) and TM (red) modes. The black dashed line denotes the functioning frequency ${fa}/c=0.277$. **e** EFSs of TE (blue) and TM (red) modes at ${fa}/c=0.277$. **f** Angle of refraction ${\theta }_{r}$ at the interface between free space and MM as a function of the incident angle ${\theta }_{i}$ for TE (blue) and TM (red) polarizations

The proposed MM is a slab composed of periodically stacked ABA dielectric layer structures along the *z* direction, as illustrated in Fig. 2a. The unit of the MM is arranged in a symmetric form, in this way the MM can be strictly homogenized as an effective medium^31,32^. We note that spatiotemporal dispersions or nonlocal responses in ABA structures have enabled anti-reflection coatings with full-polarization and omnidirectional impedance matching properties, also known as universal impedance matching layers^23^. Here, the lattice constant is set to be *a*. We assume that dielectric A is anisotropic and the principle axes are along the coordinate axes. Since one of our goals is wide-angle zero reflection, we consider a special scenario where A and B have the same Brewster angle for TM-polarized waves^33,34^. In other words, the Brewster angle ${\theta }_{{\rm{b}}}=\arctan \sqrt{{\varepsilon }_{B}}=\arcsin \sqrt{\frac{{\varepsilon }_{{Az}}{\varepsilon }_{{Ax}}-{\varepsilon }_{{Az}}}{{\varepsilon }_{{Az}}{\varepsilon }_{{Ax}}-1}\,}$^35^. Here, ${\varepsilon }_{B}$ is the relative permittivity of dielectric B, and ${\varepsilon }_{{Ax}}={\varepsilon }_{{Ay}}$ and ${\varepsilon }_{{Az}}$ are the relative permittivities of dielectric A along the *x* (or *y*) and *z* directions, respectively. Therefore, we arrive at the following condition (see Supplementary Information section 1):

1 $${\varepsilon }_{B}\left({\varepsilon }_{{Az}}-1\right)={\varepsilon }_{{Az}}\left({\varepsilon }_{{Ax}}-1\right)$$

The condition of Eq. (1) ensures perfect impedance matching at a single angle for TM polarization, i.e., the Brewster angle, which is a pre-condition of omnidirectional impedance matching. Interestingly, by further exploring the thicknesses of A and B layers (${d}_{A}$ and ${d}_{B}$), we find that it is possible to realize near-zero reflection from 0 to 89° for both polarizations, i.e., full-polarization omnidirectional Brewster effect. The design strategy is summarized in Supplementary Information section 1. For instance, we consider a case of ${\varepsilon }_{B}=25$ and ${\varepsilon }_{{Ax}}=2$. From Eq. (1), we have ${\varepsilon }_{{Az}}=25/24$. By assuming ${d}_{A}=\left(a-{d}_{B}\right)/2$ and ${d}_{B}=0.3375a$, the transmittance, calculated by transfer matrix method^36^, is >99% at the normalized frequency ${fa}/c=0.277$ for a large range of incident angles 0–86° and both TE and TM polarizations, as shown in Fig. 2b. Here, *f* is the frequency and *c* is the speed of light in free space. These results are verified again by the finite-element software COMSOL Multiphysics. Interestingly, the near-total transmission is independent of the number of the unit cells *N*, indicating this is a result of impedance matching rather than Fabry-Perot resonances that usually vary with the thickness of a slab. Figure 2c shows the wave impedance of the MM obtained from eigenstates (see Supplementary Information section 2), which indeed matches perfectly with that of free space for all incident angles and both polarizations.

In Fig. 2d, we plot the calculated band structures of the dielectric MM for TE (blue) and TM (red) modes by using COMSOL Multiphysics, where the functioning frequency is denoted by the black dashed line. Interestingly, at this frequency, the equal-frequency surfaces (EFSs) in the three-dimensional *k*-space for TE (blue) and TM (red) modes are both ellipsoids centered around the *Z* point, and they coincide with each other, as shown in Fig. 2e. Since the refraction phenomenon is determined by the shape of EFSs, the coincidence of EFSs for TE and TM modes shows that the refractive index of the MM is polarization-independent. In Fig. 2f, we plot the angle of refraction ${\theta }_{r}$ for a wave to incident on the dielectric MM at the incident angle ${\theta }_{i}$, which clearly shows polarization-independence.

**Fig. 3:**
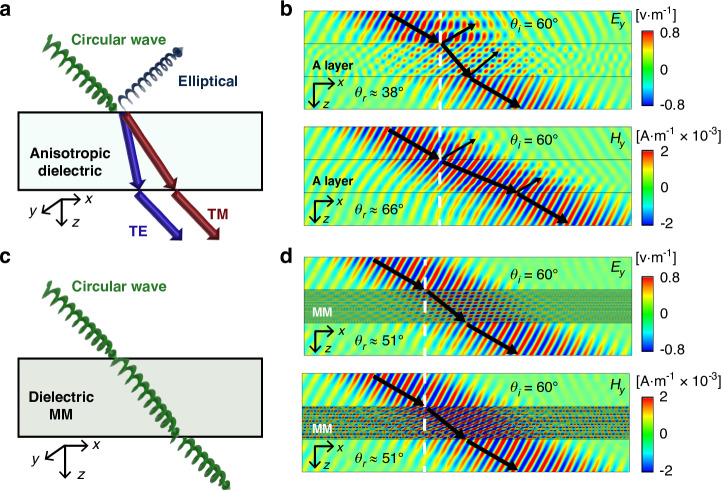
Effective self-duality-induced birefringence-free anisotropy. Illustration of circularly polarized waves incident upon (**a**) a conventional anisotropic dielectric slab, (**c**) an anisotropic pure-dielectric MM slab. Simulated distributions of ${E}_{y}$ (upper) and ${H}_{y}$ (lower) for a circularly polarized Gaussian beam incident upon (**b**) an anisotropic dielectric A slab with a thickness of $10a$, or (**d**) an anisotropic pure-dielectric MM slab with 10 unit cells under ${\theta }_{i}=60^{\circ}$ and ${fa}/c=0.277$. The angle of refraction ${\theta }_{r}$ within the A and MM slabs is indicated

The polarization-independent refraction indicates the disappearance of the birefringence phenomenon, which is a remarkable consequence of duality symmetry^5^ (see Supplementary Information section 3). Here, we numerically demonstrate this property. We consider a circularly polarized beam obliquely incident upon a conventional anisotropic dielectric slab, it is split into two beams of TE and TM polarizations propagating along different paths, as shown in Fig. 3a. Remarkably, such a beam splitting is prohibited in the designed dielectric MM, as shown in Fig. 3c. In Fig. 3b, d, we plot the calculated field distributions under the illumination of a Gaussian beam of circular polarization at ${\theta }_{i}=6{0}^{\circ }$, for the dielectric A and MM slabs of the same thickness (i.e., 10*a*), respectively. The ${E}_{y}$ (upper panel) and ${H}_{y}$ (lower panel) are related to TE and TM polarizations, respectively. The simulation results clearly show that the birefringence is evident in Fig. 3c, but disappears in Fig. 3d for the case of MM. More examples are demonstrated in Supplementary Information section 4.

Since the dielectric metamaterial is impedance-matched to free space for almost arbitrary incident angle and polarization. In this sense, it can be equivalently viewed as a slab of “stretched free space” in transformation optics^27^, which is absent of reflection and birefringence^27–30^. By retrieving the effective parameters of the MM with an even number of ABA composites (e.g., 2) using the transfer matrix method^36^ and combining the EFS shapes, we find that the MM operates as an effective uniform medium with ${\varepsilon }_{y}={\mu }_{x}=\frac{1}{{\mu }_{z}}={\mu }_{y}={\varepsilon }_{x}=\frac{1}{{\varepsilon }_{z}}\approx 0.71$, which can be obtained by stretching a layer of free space using transformation optics^27^ (see derivations in Supplementary Information sections 5 and 6). These unusual effective parameters perfectly explain the non-reflection and absence of birefringence regardless of the incident angle and polarization.

In the following, we demonstrate an experimental realization of this dielectric MM at the microwave frequencies. To simplify the realization of the anisotropic dielectric A, we assume the composite A layer is composed of two isotropic dielectric layers C and D stacked along the *z* direction, whose relative permittivities (thickness) are ${\varepsilon }_{C}$ (${d}_{C}$) and ${\varepsilon }_{D}$ (${d}_{D}$), respectively. The filling ratio of the component C is ${f}_{C}={d}_{C}/\left({d}_{C}+{d}_{D}\right)$. At the deep subwavelength scale, we could apply the quasi-static effective medium theory^37^ for dielectric A as ${\varepsilon }_{{Ax},{\rm{eff}}}={f}_{C}{\varepsilon }_{C}+\left(1-{f}_{C}\right){\varepsilon }_{D}$ and ${\varepsilon }_{{Az},{\rm{eff}}}=\frac{{\varepsilon }_{C}{\varepsilon }_{D}}{{f}_{C}{\varepsilon }_{D}\,+\,\left(1-{f}_{C}\right){\varepsilon }_{C}}$, respectively. There is a special solution, i.e.

2 $${\varepsilon }_{C}={\varepsilon }_{B},{\varepsilon }_{D}=1\,{\rm{and}}\,{f}_{C}\in \left(0,1\right)$$

Under this condition, the Eq. (1) is valid for arbitrary values of ${\varepsilon }_{B}$ and ${f}_{C}$.

**Fig. 4:**
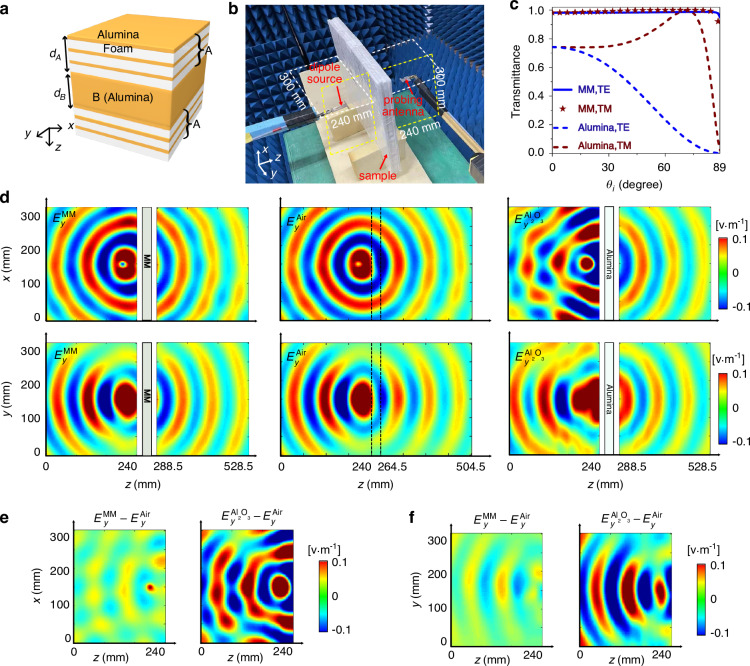
Experimental observation of full-polarization omnidirectional Brewster effect. **a** Illustration of the unit cell of the designed MM consisting of different alumina layers spaced by foam. **b** Picture of the fabricated sample with a unit cell and the measuring environment. **c** Simulated transmittance through the MM sample (solid lines and symbols) and an alumina slab of the same thickness (dashed lines) for TE (blue) and TM (red) polarizations as a function of the incident angle at 3.8 GHz. **d** Measured ${E}_{y}$ on the *xz* (upper) and *yz* (lower) planes at 3.8 GHz. The left, middle and right panel graphs denote the case of the MM sample, the case of free space and the case of the alumina sample, respectively. Scattered fields in the cases of the MM (left) and pure alumina (right) samples on the (**e**) *xz* and **f** *yz* planes on the source side

Based on Eq. (1) and (2), we have realized a sample with ${\varepsilon }_{B}={\varepsilon }_{C}=8.9$ (alumina) and ${\varepsilon }_{D}=1$ (foam). As schematically shown in Fig. 4a, each A layer is composed of three C-D units with *d*_*C*_ = 0.4 mm and *d*_*D*_ = 1.8 mm, thus each A layer has a thickness of 6.6 mm and the effective parameters of ${\varepsilon }_{{Ax}}=2.44$ and ${\varepsilon }_{{Az}}=1.19$, approximately satisfying the condition in Eq. (1). The thickness of B layer is 11.3 mm. The operating frequency is 3.8 GHz. Figure 4b displays the photograph of the fabricated sample. Figure 4c shows the transmittance through this MM is near 100% for both TE (blue) and TM (red) polarizations, regardless of the incident angle and polarization. For contrast, the transmittance through a pure alumina slab of the same thickness is apparently much lower.

In the microwave experiment, we investigate the reflection and transmission of a dipole source (polarized along the *y* direction) placed nearby the MM sample at a distance of 39.5 mm (to the center of the sample). A probing antenna is attached to a Keysight PNA-X 5242A network analyzer to measure the near-field electric fields, as shown in Fig. 4b. The scanning areas are of 300 × 240 mm^2^ (the areas marked by dashed lines), located on the *xz* and *yz* planes on the both sides of the MM sample, with a distance of 30 mm from the source antenna. The measured electric-field distributions are shown in the left upper and lower panel graphs of Fig. 4d. We note that there is an air gap of 12 mm between the MM slab and the scanning area on each side, because of the size of the probing antenna itself. The measured results clearly show cylindrical wave pattern on the *xz* plane and dipole radiation pattern on the *yz* plane. The absence of the interference patterns indicates that the reflection is zero. For comparison, the measurement is performed again for free space without the MM sample (middle). The field patterns on the source side are almost the same in these two cases, which proves that the MM slab has no reflection under the illumination of the nearby dipole source. In the forward radiation patterns, there is a phase difference in the above two cases, which is caused by the shift of EFCs to the *Z* point in the MM^29^. Finally, we measured the electric field distributions by replacing the MM sample with an alumina slab of the same thickness (right). There is clear reflection from the alumina slab. We retrieved the scattered field distributions, i.e., ${E}_{y}^{{\rm{MM}}}-{E}_{y}^{{\rm{Air}}}$ and ${E}_{y}^{{{\rm{Al}}}_{2}{{\rm{O}}}_{3}}-{E}_{y}^{{\rm{Air}}}$, on the *xz* and *yz* planes on the source side, which are shown in Fig. 4e, f, respectively. The average intensity of the scattered field from the MM sample is over 20 times smaller than that from the alumina slab. The residual reflection of the MM sample mainly originates from the imperfection of the fabrication and inevitable material loss. The simulation results and more detailed experimental results are presented in Supplementary Information section 7.

It is worth noting that the designed MM exhibits a relatively broad bandwidth of high transmittance (>0.9). Specifically, at normal incidence, the bandwidth for achieving a transmittance of 0.9 spans 0.9 GHz. Despite the reduction in bandwidth for TE polarization as the incident angle ${\theta }_{i}$ increases, the MM maintains a bandwidth of 0.38 GHz at ${\theta }_{i}=60^{\circ}$. Notably, the bandwidth expands for TM polarization as the incident angle increases, peaking at the predetermined Brewster angle. This unique property enhances the wave-transport performance for TM polarization. We observe that the bandwidth of 0.9 transmittance exceeds 0.9 GHz for incident angles up to 80° (see Supplementary Information section 7), meeting requirements for applications such as 5 G communication. Furthermore, the bandwidth could be further widened through introducing more degrees of freedom (e.g., additional components) and appropriate frequency dispersions to the permittivities of the components.

**Fig. 5:**
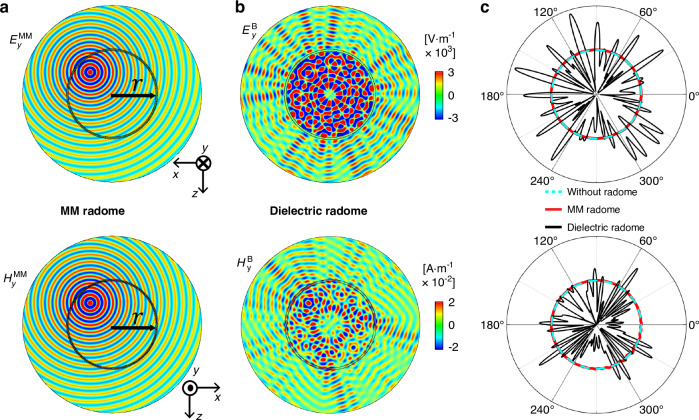
Totally wave-transparent MM radomes. Simulated distributions of electric (upper) and magnetic (lower) fields radiated from out-of-plane electric (upper) and magnetic (lower) monopolar sources, placed off-center within a circular (**a**) MM, (**b**) dielectric radome. The MM radome is composed of a single ABA unit, adopted from Fig. 2, and the dielectric radome is made of pure dielectric B. The two radomes have the same total thickness. **c** Far-field radiation patterns without any radome (cyan), with a MM radome (red), with a dielectric radome (black) for the cases of out-of-plane electric (upper) and magnetic (lower) monopolar sources. The functioning frequency is 8.30 GHz

The effective self-dual characteristic and full-polarization omnidirectional Brewster effect of the designed pure-dielectric MM are highly desired in the applications of radomes, which protect the radars but at the same time allow electromagnetic signals to be emitted from or received by the radar without any distortion or attenuation. Figure 5 shows an example to demonstrate the feasibility. Here, we examine the radiation patterns of a point source positioned off-center within a circular MM radome (Fig. 5a). The MM radome comprises a single ABA unit, adopted from the theoretical model in Fig. 2 with *a* = 10 mm. Two more examples of MM radomes comprising 3 and 4 layers of ABA units are demonstrated in Supplementary Information section 8. The radome has a radius of *r* = 217 mm, and the functioning frequency is 8.30 GHz. The upper and lower panel graphs show the distributions of electric field ${E}_{y}^{{\rm{MM}}}$ and magnetic field ${H}_{y}^{{\rm{MM}}}$ radiated from out-of-plane electric and magnetic monopolar sources, respectively, revealing nearly undisturbed cylindrical wave patterns. The MM radome exhibits wave transparency for both polarizations, just like free space, as further evidenced in the far-field radiation patterns in Fig. 5c. By contrast, significant distortions in the radiation patterns are observed when the MM radome is replaced by a dielectric radome of the same dimensions, as shown by the distributions of electric field ${E}_{y}^{{\rm{B}}}$ and magnetic field ${H}_{y}^{{\rm{B}}}$ in Fig. 5b, as well the far-field radiation patterns in Fig. 5c. Here, the dielectric material is chosen as dielectric B. These results underscore the viability of the self-dual MM as a radome that is totally wave-transparent to electromagnetic signals, without causing any distortion or attenuation.

Besides the radomes, the proposed MMs could also be used for full-parameter transformation-optics devices, without the need for complicated magnetic responses. In addition, the MMs could also find applications in reflectionless dielectric metasurfaces. Since the phase of transmitted waves can be tuned by engineering dispersion curves, reflectionless gradient metasurfaces consisting of reflectionless units with different transmission phases could be constructed for high-efficiency meta-devices like metalenses.

## Discussion

It is exciting that the self-dual MM realized here is pure dielectric and does not involve any magnetic or metal ingredients (see Supplementary Information sections 9 and 10). This valuable property opens the possibility of achieving self-duality at relatively high frequencies, such as infrared frequencies, by using optical materials such as silicon. An example working at the infrared frequency is demonstrated in Supplementary Information section 11. Another significant advantage of pure dielectric design is that loss can be negligible.

It is noteworthy that full-polarization omnidirectional impedance matching can also be achieved by using the method of universal impedance matching layers, which are special anti-reflection coatings exhibiting spatiotemporal dispersions or nonlocal responses^23^. Our MMs with effective self-duality symmetry are wave-transparent anisotropic materials by themselves, with polarization-independent refractive properties in accordance with the concept of “stretched free space” in transformation optics. Therefore, our work provides a low-loss and feasible platform for perfect transformation-optics devices.

Despite the recent significant progress achieved in the novel Brewster effect of artificial materials, such as the generalized Brewster effect^29,38–45^, plasmonic Brewster effect^46–49^ and anomalous Brewster effect^50–52^, etc., the full-polarization and omnidirectional Brewster effect with self-duality distinguishes itself by providing an unprecedented approach for eliminating all the undesired wave phenomena induced by reflection, regardless of the wavefront and polarization morphologies. This essential feature holds potential for realizing novel wave-transparent devices, such as perfect solid radomes resembling free space.

## Materials and methods

### Simulations

Numerical simulations in this work are performed by using the commercial finite-element simulation software COMSOL Multiphysics. In the calculation of transmittance in Figs. 2b and 4c, Floquet periodic boundaries are set as the boundaries on the *xz* and *yz* planes, and port is set on the *xy* plane to excite the incident wave. The band structures and EFSs in Fig. 2d, e are calculated using a continuum Floquet eigensolver with Floquet periodic boundary conditions. The eigen-frequencies can be calculated for given Bloch wave vectors. In Fig. 3b, d, the Gaussian beam of circular polarization is excited through the background wave in COMSOL Multiphysics, and the bottom boundary is set as the perfect matched layer to absorb the transmitted waves. The fields and far-field radiation patterns in Fig. 5a–c are obtained by placing out-of-plane electric and magnetic monopolar sources inside radomes. The outermost boundary is set as the perfect matched layer to absorb the radiated waves.

### Experiments

The experiment was performed in an anechoic chamber with a Keysight PNA-X 5242A network analyzer. A dipole antenna is used as the signal source to generate electromagnetic waves, and another one is used to probe the near-field electric fields. The input signal has a power of 0 dBm and passes through a 23 dBm amplifier. Both the dipole antennas are placed horizontally along *y* direction, therefore only the ${E}_{y}$-distributions are detected here. The source antenna is placed at a distance of 39.5 mm from the center of the fabricated MM sample. The detecting probe is mounted to a computer controlled translational stage and scan in a precision of 3 mm per step. The scanning areas are located on both the ${xz}$ and ${yz}$ planes before and after the MM sample. The scanning areas are 300 × 240 mm^2^ each, which are marked by dashed lines in Fig. 4b. Each scanning area has a distance of 30 mm from the source antenna.

## Supplementary information

Supplementary Information for Dielectric Metamaterials with Effective Self-duality and Full-polarization Omnidirectional Brewster Effect
