# Supplementary material for: Dielectric metamaterials with effective self-duality and full-polarization omnidirectional brewster effect
Source: Light Sci Appl. 2024 Sep 20;13:262. doi: 10.1038/s41377-024-01605-z (PMC11412996; doi:10.1038/s41377-024-01605-z)
Supplement: Supplementary file 1 — Supplementary Information for Dielectric Metamaterials with Effective Self-duality and Full-polarization Omnidirectional Brewster Effect [file 41377_2024_1605_MOESM1_ESM.docx]

Supplementary Information for

**Dielectric Metamaterials with Effective Self-duality and Full-polarization Omnidirectional Brewster Effect**

Hao Luo^1#^, Jie Luo^2,^*^#^, Zhihui Zhang^3,#^, Chao Wu^4^, Quan Li^4^, Wei Liu^5^, Ruwen Peng^1^, Mu Wang^1^, Hongqiang Li^4,^*, Yun Lai^1,^*

^1^National Laboratory of Solid State Microstructures, School of Physics, and Collaborative Innovation Center of Advanced Microstructures, Nanjing University, Nanjing 210093, China

^2^Institute of Theoretical and Applied Physics, School of Physical Science and Technology, Soochow University, Suzhou 215006, China

^3^School of Physics Science and Engineering, Tongji University, Shanghai 200092, China

^4^College of Electronic and Information Engineering, Tongji University, Shanghai 200092, China

^5^College for Advanced Interdisciplinary Studies, National University of Defense Technology, Changsha, Hunan 410073, China

*Corresponding authors: Yun Lai (laiyun@nju.edu.cn); Jie Luo (luojie@suda.edu.cn); Hongqiang Li ([hqlee@tongji.edu.cn](mailto:hqlee@tongji.edu.cn))

^#^These authors contributed equally: Hao Luo, Jie Luo, Zhihui Zhang

1. [Predetermined Brewster’s angle in the pure-dielectric metamaterial](#_Brewster_angle_of)
2. Wave impedance of the all-dielectric metamaterial retrieved from eigenfields
3. Electromagnetic duality transformation and polarization independence
4. Reflection-less and birefringence-free characteristics for arbitrary wavefront and polarization
5. Retrieval of effective parameters of the pure-dielectric metamaterial
6. [Effective anisotropy and equivalence of “stretched free space”](#_Effective_parameters_of)
7. Experimental setup, measurement methods and supplemental simulation results
8. **More examples of MM radomes**
9. **A practical realization of microwave self-dual metamaterial**
10. **More examples of self-dual pure-dielectric metamaterials**
11. **A practical realization of infrared self-dual metamaterial based on silicon**

References

## Predetermined Brewster’s angle in the pure-dielectric metamaterial

We assume a pure-dielectric metamaterial (MM) composed of layers of anisotropic dielectric A and isotropic dielectric B. It is known that the Brewster angle on the air-isotropic B interface for transverse-magnetic (TM) polarization is $\arctan\sqrt{\varepsilon_{B}}$, where $\varepsilon_{B}$ is the relative permittivity. On the other hand, the Brewster angle for the anisotropic dielectric A characterized by a relative permittivity tensor of $\left( \begin{matrix} \varepsilon_{Ax} & & \\ & \varepsilon_{Ay} & \\ & & \varepsilon_{Az} \end{matrix} \right)$ can be derived by imposing a zero Fresnel’s reflection coefficient. Here, we assume that $\varepsilon_{Ax}=\varepsilon_{Ay}$. The reflection coefficient at the air-anisotropic A interface for TM polarization is expressed as^1^,

$r=\frac{\sqrt{\varepsilon_{Ax}\varepsilon_{Az}}\cos\theta_{i}-\sqrt{\varepsilon_{Az}-\sin^{2} \theta_{i}}}{\sqrt{\varepsilon_{Ax}\varepsilon_{Az}}\cos\theta_{i}+\sqrt{\varepsilon_{Az}-\sin^{2} \theta_{i}}}$ (S1)

The condition of $r=0$ leads to the Brewster angle of $\arcsin\sqrt{\frac{\varepsilon_{Az}\varepsilon_{Ax}-\varepsilon_{Az}}{\varepsilon_{Az}\varepsilon_{Ax}-1}}$. When the dielectric A and B possess the same Brewster angle, we obtain,

$\varepsilon_{B}\left( \varepsilon_{Az}-1 \right)=\varepsilon_{Az}\left( \varepsilon_{Ax}-1 \right)$ (S2)

Under this circumstance, we can always expect the perfect transmission on the dielectric MM for TM-polarized waves under the predetermined Brewster angle.

In order to achieve full-polarization omnidirectional Brewster effect within the MMs, the electromagnetic and geometrical parameters of the MMs need to be further optimized. The condition in Eq. (S2) indicates that there are four independent parameters, namely $\varepsilon_{Ax}(=\varepsilon_{Ay})$, $\varepsilon_{B}$, $d_{A}$, $d_{B}$, for optimization at a chosen working frequency. During the optimization, there are two goals: 1) Omnidirectional impedance matching between the ABA structure and free space; 2) The same dispersion curves for TE and TM polarizations.

To explore the influences of the four parameters $\varepsilon_{Ax}(=\varepsilon_{Ay})$, $\varepsilon_{B}$, $d_{A}$, $d_{B}$ on the impedance and dispersions of MMs, we systematically vary each parameter individually while keeping other parameters unchanged at a selected frequency (or wavelength $\lambda$). Our numerical simulation results reveal that the values of the four parameters have weak influences on the coincidence of band structures for TE and TM polarizations due to their inherent overlap at the center of the Brillouin zone. Instead, they predominantly impact the wave impedance of the MMs. Specifically, we observe that achieving impedance matching necessitates $\varepsilon_{B}>\varepsilon_{Ax}$, and a larger contrast generally leads to better performance. In addition, the component B approximately satisfies the condition $\sqrt{\varepsilon_{B}}d_{B}\sim0.5\lambda$, leading to destructive interference of reflected waves on component B. The thickness of component A (i.e., $d_{A}$) varies in the range of $0.8d_{B}\sim0.9d_{B}$ for optimal impedance matching. Consequently, this significantly reduces the parameter this. Subsequent comprehensive optimization of all four parameters enables the realization of the full-polarization omnidirectional Brewster effect.

## Wave impedance of the all-dielectric metamaterial retrieved from eigenfields

The dispersion of the dielectric MM can be obtained based on eigenmode analysis by using the finite-element software COMSOL Multiphysics. The eigen-frequency for any chosen Bloch wave vector $\mathbf{k}_{B}=k_{x}\hat{x}+k_{z}\hat{z}$ can be calculated, so as to obtain the dispersion characteristic. In addition, the effective wave impedance can be obtained from the eigen-fields as^2^,

$Z_{x,\mathrm{eff}}^{\mathrm{TE}}=\frac{\left\langle E_{y} \right\rangle_{xy}}{\left\langle H_{z} \right\rangle_{xy}} ,$ $Z_{z,\mathrm{eff}}^{\mathrm{TE}}=\frac{\left\langle E_{y} \right\rangle_{xy}}{\left\langle H_{x} \right\rangle_{xy}}$ , $Z_{x,\mathrm{eff}}^{\mathrm{TM}}=\frac{\left\langle E_{z} \right\rangle_{xy}}{\left\langle H_{y} \right\rangle_{xy}}$ , and $Z_{z,\mathrm{eff}}^{\mathrm{TM}}=\frac{\left\langle E_{x} \right\rangle_{xy}}{\left\langle H_{y} \right\rangle_{xy}}$ (S3)

where $\left\langle\ldots\right\rangle_{xy}$ denotes the average of eigenfields on the $xy$ surface of the MM unit cell.

This approach is valid based on the following assumptions: 1) Single-mode approximation, i.e., only one eigenmode is excited; 2) The amplitudes of eigen-fields on the incident boundary are almost constant, and the phases approximately obey the trigonometric functions; 3) The eigen-electric and eigen-magnetic fields are nearly in phase at the incident boundary. Although these premises are seemingly stringent, it turns out that most eigenmodes of the first few bands can indeed satisfy these requirements**.**

## Electromagnetic duality transformation and polarization independence

Electromagnetic duality transformation is a transformation where the roles of electric and magnetic fields are mixed, and is expressed as^3^

$\left( \begin{aligned} \mathbf{E} \\ Z_{0}\mathbf{H} \end{aligned} \right)\to\left( \begin{aligned} \mathbf{E}' \\ Z_{0}\mathbf{H}' \end{aligned} \right)=T\left( \xi\right)\left( \begin{aligned} \mathbf{E} \\ Z_{0}\mathbf{H} \end{aligned} \right)$ (S4a)

$\left( \begin{aligned} Z_{0}\mathbf{D} \\ \mathbf{B} \end{aligned} \right)\to\left( \begin{aligned} Z_{0}\mathbf{D}' \\ \mathbf{B}' \end{aligned} \right)=T\left( \xi\right)\left( \begin{aligned} Z_{0}\mathbf{D} \\ \mathbf{B} \end{aligned} \right)$ (S4b)

where $Z_{0}$ is the characteristic impedance of free space. $T\left( \xi\right)$ is the duality transformation matrix as

$T\left( \xi\right)=\left( \begin{matrix} \cos\xi& -\sin\xi\\ \sin\xi& \cos\xi\end{matrix} \right)$ (S5)

where $\xi$ is a real transformation angle. The duality symmetry requires that the field $(\mathbf{E}^{'}, \mathbf{H}'$) is a solution of the Maxwell equations in the absence of charges and currents, if the field $(\mathbf{E}, \mathbf{H})$ is a solution. Before the duality transformation, the constitutive relation is given by

$\left( \begin{aligned} Z_{0}\mathbf{D} \\ \mathbf{B} \end{aligned} \right)=c^{-1}\left( \begin{matrix} \varepsilon_{r} & 0 \\ 0 & \mu_{r} \end{matrix} \right)\left( \begin{aligned} \mathbf{E} \\ Z_{0}\mathbf{H} \end{aligned} \right)$ (S6)

where $c$ is the speed of light in free space. $\varepsilon_{r}$ and $\mu_{r}$ are the relative permittivity and permeability, respectively. Combing Eqs. (S4) and (S6), the constitutive relation of Eq. (S4b) after the duality transformation becomes

$\left( \begin{aligned} Z_{0}\mathbf{D}' \\ \mathbf{B}' \end{aligned} \right)=c^{-1}\left( \begin{matrix} \varepsilon_{r}+\left( \mu_{r}-\varepsilon_{r} \right)\sin^{2} \xi& \frac{1}{2}\left( \varepsilon_{r}-\mu_{r} \right)\sin\left( 2\xi\right) \\ \frac{1}{2}\left( \varepsilon_{r}-\mu_{r} \right)\sin\left( 2\xi\right) & \varepsilon_{r}+\left( \mu_{r}-\varepsilon_{r} \right)\cos^{2} \xi\end{matrix} \right)\left( \begin{aligned} \mathbf{E}' \\ Z_{0}\mathbf{H}' \end{aligned} \right)$ (S7)

Equation (S7) indicates that the duality transformation general requires bianisotropic materials within which electric and magnetic fields are coupled with each other. Intriguingly, when $\varepsilon_{r}=\mu_{r}$, Eq. (S7) becomes

$\left( \begin{aligned} Z_{0}\mathbf{D}' \\ \mathbf{B}' \end{aligned} \right)=c^{-1}\left( \begin{matrix} \varepsilon_{r} & 0 \\ 0 & \mu_{r} \end{matrix} \right)\left( \begin{aligned} \mathbf{E}' \\ Z_{0}\mathbf{H}' \end{aligned} \right)$ (S8)

We see from Eq. (S8) that the constitutive relation is unchanged after the duality transformation, independent of $\xi$, for materials satistifying $\varepsilon_{r}=\mu_{r}$, which are usually said to be self-dual. When setting $\xi=\pi/2$, we obtain the typical exchange $\mathbf{E}\to Z_{0}\mathbf{H}\mathbf{'}$ and $Z_{0}\mathbf{H}\to\mathbf{-E}\mathbf{'}$ in self-dual materials, which leads to the polarization-independent electromagnetic properties.

## Reflection-less and birefringence-free characteristics for arbitrary wavefront and polarization

In Fig. 2 in the main text, a theoretical model of a pure-dielectric MM exhibiting the full-polarization and near-omnidirectional Brewster effect is demonstrated, which allows near-perfect transmission of waves for arbitrary wavefront and polarization. For further verification, here we examine the radiation of an electric dipole source (oriented along the $y$ direction) placed above the dielectric MM slab at a distance of $0.6\lambda_{0}$ ($\lambda_{0}$ is the wavelength in free space), as shown schematically in Fig. S1(a). Figure S1(b) shows the simulated distribution of the electric field $E_{y}$ for the dielectric MM slab consisting of one layer of unit cell. In Figs. S1(c) and S1(d), we also plot the snapshots of $E_{y}$ on $xz$ plane which cut through the dipole, and $H_{x}$ on $yz$ plane where the dipole lies on, respectively. In the simulation results, a well-defined cylindrical wave pattern is observed on $xz$ plane [Fig. S1(c)] and a dipole radiation pattern is observed on $yz$ plane [Fig. S1(d)]. In both cases, there is no clear interference pattern induced by reflection, confirming the full-polarization omnidirectional Brewster effect within the dielectric MM.

**
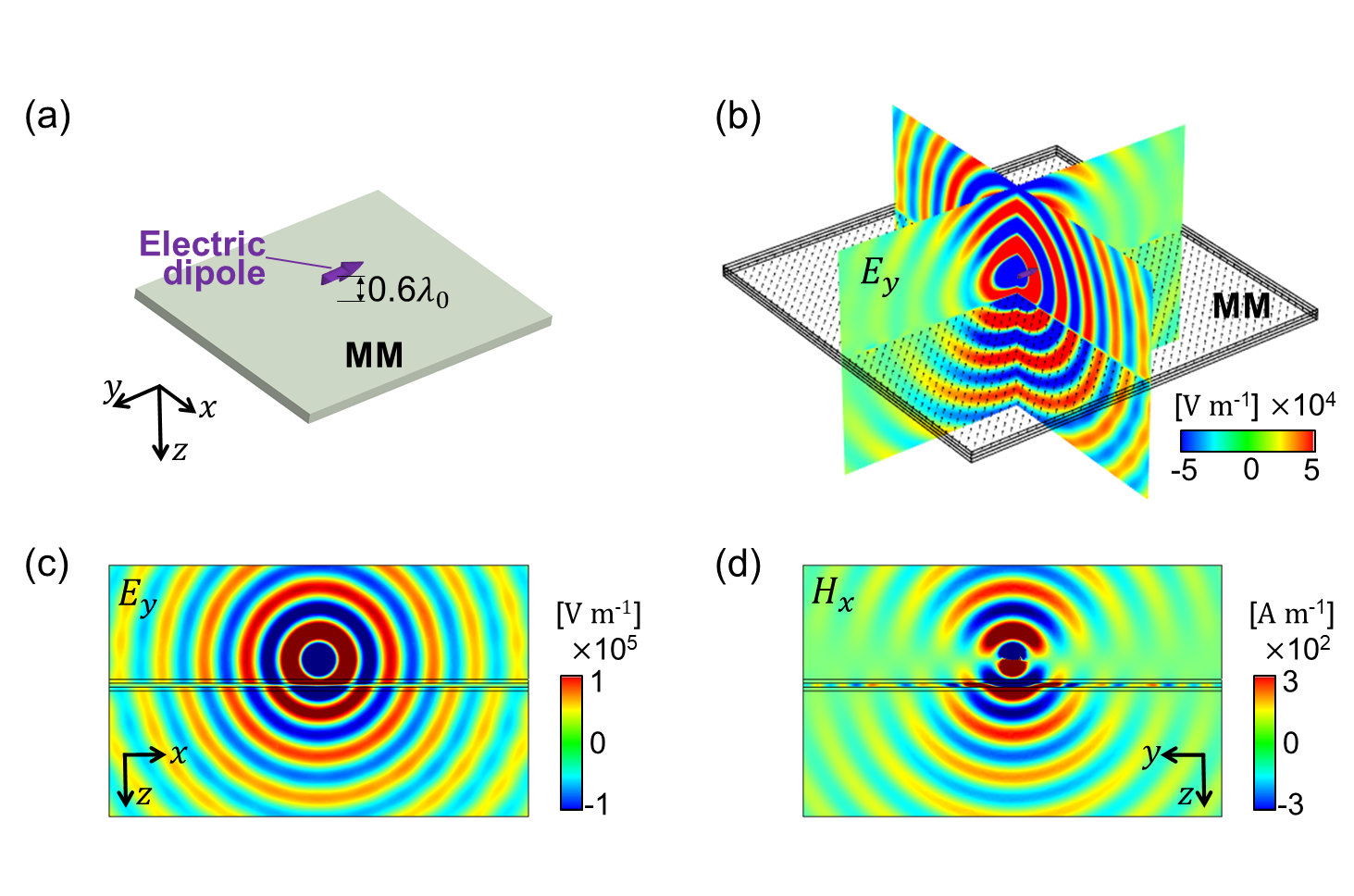
**

**Fig. S1.** (a) Illustration of an electric dipole oriented along $y$ direction placed above the dielectric MM slab at a distance of $0.6\lambda_{0}$. (b) Simulated $E_{y}$-distribution for the MM slab consisting of one unit cell. [(c) and (d)] Snapshots of (c) $E_{y}$ on $xz$ plane cutting the dipole, (d) $H_{x}$ on $yz$ plane the dipole lies on taken from (b).

In Fig. 3 in the main text, the unique birefringence-free characteristic of the dielectric MM is demonstrated by examining the refraction behaviors of TE and TM polarized waves. Due to the artificial duality symmetry, the responses for TE and TM polarizations are exactly the same, including the refraction behaviors, and thus the birefringence disappears. Here, we’d like to show more examples. Figures S2(a)-S2(c) show the simulated field distributions on the MM slab (10 unit cells) under the illumination of a circularly polarized Gaussian beam at $30^{\circ}$, $50^{\circ}$ and $70^{\circ}$, respectively. The upper and lower panel graphs show, respectively, the distributions of $E_{y}$ and $H_{y}$ on $xz$ plane. We see that the outgoing waves with TE- ($E_{y}$) and TM- ($H_{y}$) polarizations transmit through the MM slab at the same position, with no reflection, irrespective of the incident angles. These results further demonstrate the reflection-less and birefringence-free characteristics of the pure-dielectric MM.

Moreover, we consider Gaussian beams with narrow waist, which spread along the propagation path. Figures S3(a) and S3(b) show the simulated field distributions on the MM slab (10 unit cells) under the illumination of TE- (upper) and TM- (lower) polarized Gaussian beams at the incident angles of $0^{\circ}$ and $45^{\circ}$, respectively. We see that the reflection is absent, and the outgoing waves with TE- and TM- polarizations transmit through the MM slab at the same position. These results further demonstrate the reflection-less and birefringence-free characteristics for arbitrary wavefront and polarization.


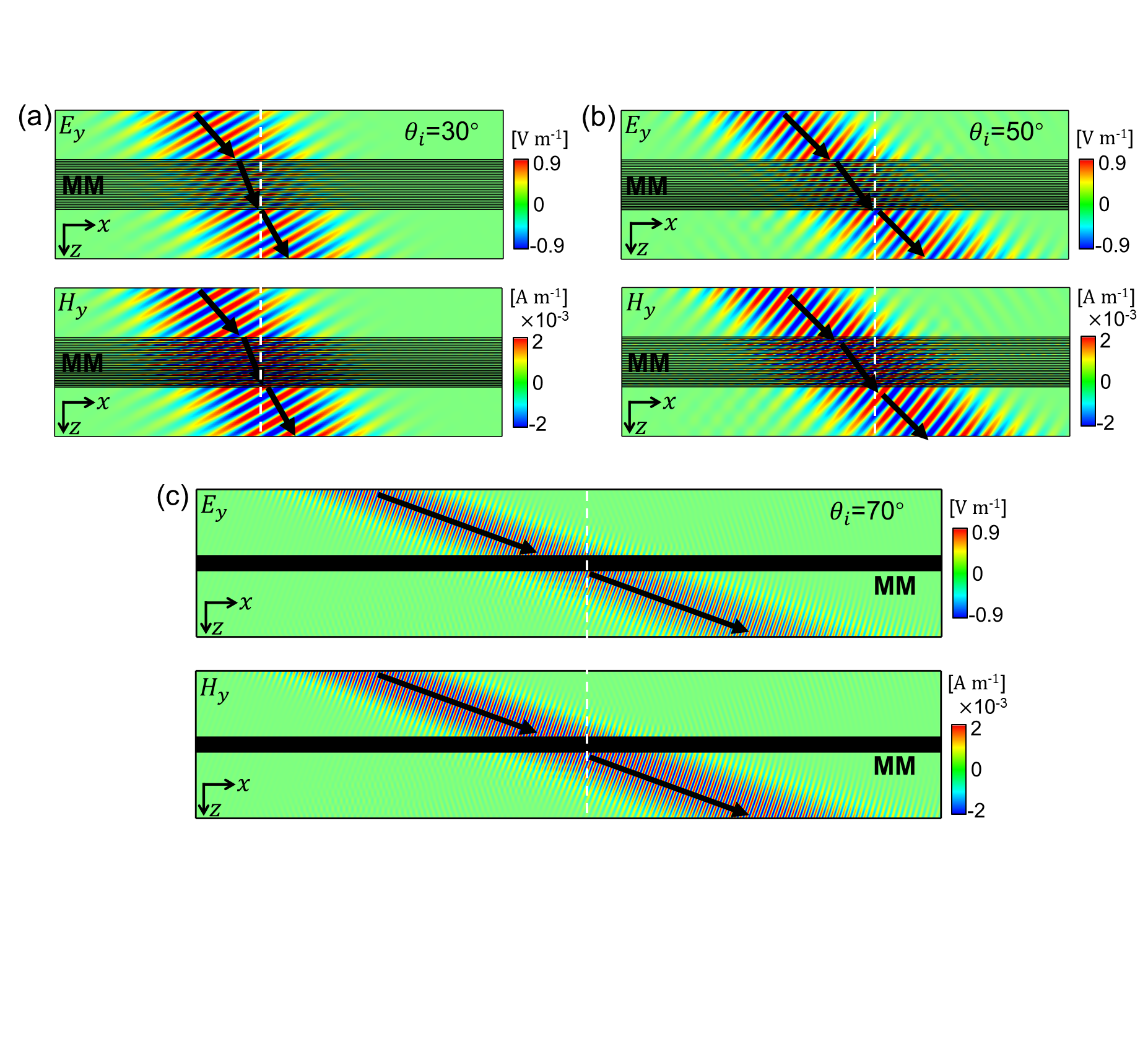


**Fig. S2.** Distributions of $E_{y}$ (upper) and $H_{y}$ (lower) on $xz$ plane when a circularly polarized Gaussian beam is incident onto the dielectric MM slab composed of 10 unit cells under the incident angles of (a) $30^{\circ}$, (b) $50^{\circ}$, (c) $70^{\circ}$.


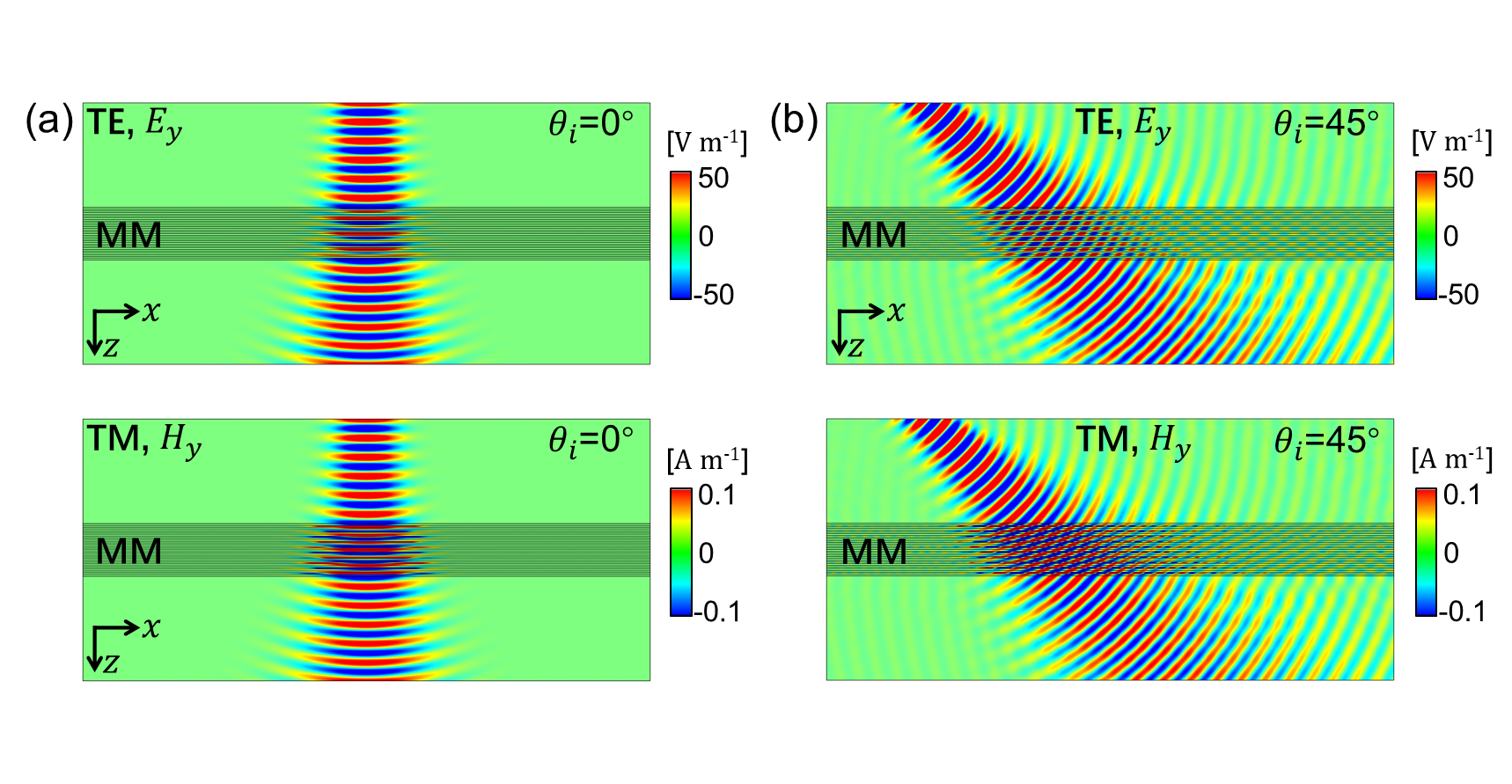


**Fig. S3.** Distributions of $E_{y}$ (upper) and $H_{y}$ (lower) on $xz$ plane when TE- (upper) and TM- (lower) polarized Gaussian beams with narrow waist incident onto the dielectric MM slab composed of 10 unit cells at the incident angles of (a) $0^{\circ}$ and (b) $45^{\circ}$.

## Retrieval of effective parameters of the pure-dielectric metamaterial

The effective parameters of the pure-dielectric MM are retrieved through matching the reflection and transmission coefficients between the MM and an effective medium based on the transfer matrix method^4,5^, as we will show as follows.

First, we consider the MM consisting of ABA units illuminated by a TE-polarized plane wave (electric field in the $y$ direction) under the incident angle of $\theta_{i}$ from free space. According to the transfer matrix method ^5^, the total transfer matrix of an ABA unit can be expressed as,

$M^{\mathrm{TE}} =M_{A}^{\mathrm{TE}}M_{B}^{\mathrm{TE}}M_{A}^{\mathrm{TE}}=\left( \begin{matrix} M_{11}^{\mathrm{TE}} & M_{12}^{\mathrm{TE}} \\ M_{21}^{\mathrm{TE}} & M_{22}^{\mathrm{TE}} \end{matrix} \right)$ (S9)

where $M_{A,B}^{\mathrm{TE}}=\left( \begin{matrix} \cos x_{A,B} & \left( ia_{A,B}/b_{A,B} \right)\sin x_{A,B} \\ \left( ib_{A,B}/a_{A,B} \right)\sin x_{A,B} & \cos x_{A,B} \end{matrix} \right)$ is the transfer matrix of the A (B) layer. Here, $a_{A}^{2}=\mu_{A}$, $a_{B}^{2}=\mu_{B}$, $b_{A}^{2}=\varepsilon_{Ay}-\frac{\sin^{2} \theta_{i}}{\mu_{A}}$, $b_{B}^{2}=\varepsilon_{B}-\frac{\sin^{2} \theta_{i}}{\mu_{B}}$, and $x_{A,B}=a_{A,B}b_{A,B}k_{0}d_{A,B}$. $k_{0}$ is the wave number in free space. $M_{ij}^{\mathrm{TE}}$ ($i=1,2$, $j=1,2$) is the element of the matrix $M^{\mathrm{TE}}$ in the $i$-th row and $j$-th column. Then, the reflection and transmission coefficients for TE polarization can be derived as ^5^,

$r^{\mathrm{TE}} =\frac{\left( \eta_{0}^{\mathrm{TE}}M_{22}^{\mathrm{TE}}-\eta_{0}^{\mathrm{TE}}M_{11}^{\mathrm{TE}} \right)-\left( \left( \eta_{0}^{\mathrm{TE}} \right)^{2}M_{12}^{\mathrm{TE}}-M_{21}^{\mathrm{TE}} \right)}{\left( \eta_{0}^{\mathrm{TE}}M_{22}^{\mathrm{TE}}+\eta_{0}^{\mathrm{TE}}M_{11}^{\mathrm{TE}} \right)-\left( \left( \eta_{0}^{\mathrm{TE}} \right)^{2}M_{12}^{\mathrm{TE}}+M_{21}^{\mathrm{TE}} \right)}$ (S10a)

$t^{\mathrm{TE}}=\frac{2\eta_{0}^{\mathrm{TE}}}{\left( \eta_{0}^{\mathrm{TE}}M_{22}^{\mathrm{TE}}+\eta_{0}^{\mathrm{TE}}M_{11}^{\mathrm{TE}} \right)-\left( \left( \eta_{0}^{\mathrm{TE}} \right)^{2}M_{12}^{\mathrm{TE}}+M_{21}^{\mathrm{TE}} \right)}$ (S10b)

with $\eta_{0}^{\mathrm{TE}}=\cos\theta_{i}$. Since the ABA unit possesses mirror symmetry, it can be proved that $M_{11}^{\mathrm{TE}}=M_{22}^{\mathrm{TE}}$ and $M_{11}^{\mathrm{TE}}M_{22}^{\mathrm{TE}}-M_{12}^{\mathrm{TE}}M_{21}^{\mathrm{TE}}=1$ based on Eq. (S9) ^6,7^. Moreover, in the absence of material loss or gain, $M_{11}^{\mathrm{TE}}$and $M_{22}^{\mathrm{TE}}$ are real values, while $M_{12}^{\mathrm{TE}}$ and $M_{21}^{\mathrm{TE}}$ are imaginary values. These properties indicate that such an ABA unit can be regarded as a uniform effective medium layer ^6,7^, whose transfer matrix can be expressed as,

$M^{\mathrm{TE}}=\binom{\begin{matrix} \cos x & \left( ia/b \right)\sin x \end{matrix}}{\begin{matrix} \left( ib/a \right)\sin x & \cos x \end{matrix}}$ (S11)

where $x=abk_{0}D$, $a^{2}=\mu_{x,\mathrm{eff}}$ and $b^{2}=\varepsilon_{y,\mathrm{eff}}-\frac{\sin^{2} \theta_{i}}{\mu_{z,\mathrm{eff}}}$. $D$ is the total thickness of the ABA unit, i.e., $D=2d_{A}+d_{B}$. The uniform effective medium is characterized by an effective relative permittivity tensor $\left( \begin{matrix} \varepsilon_{x,\mathrm{eff}} & & \\ & \varepsilon_{y,\mathrm{eff}} & \\ & & \varepsilon_{z,\mathrm{eff}} \end{matrix} \right)$ and an effective relative permeability tensor $\left( \begin{matrix} \mu_{x,\mathrm{eff}} & & \\ & \mu_{y,\mathrm{eff}} & \\ & & \mu_{z,\mathrm{eff}} \end{matrix} \right)$.

Such an effective medium shall exhibit the same reflection and transmission coefficients as the actual ABA unit. Since the reflection and transmission coefficients can be directly obtained using numerical software like COMSOL Multiphysics to simulate the real ABA structure, then, combined Eqs. (S10) and (S11), we find that the effective parameters shall satisfy the following conditions,

$\mu_{x,\mathrm{eff}}=-\frac{p_{1}p_{2}}{\cos\theta_{i}}\pm\sqrt{\frac{({(p_{1})}^{2}+1){(p_{2})}^{2}}{\cos^{2} \theta_{i}}}$ (S12a)

$\varepsilon_{y,\mathrm{eff}}-\frac{\sin^{2} \theta_{i}}{\mu_{z,\mathrm{eff}}}-\mu_{x,\mathrm{eff}}\cos^{2} \theta_{i}-2p_{1}p_{2}\cos\theta_{i}=0$ (S12b)

where $p_{1}=\frac{\left( \frac{\mathrm{Im}\left( r^{\mathrm{TE}} \right)\mathrm{Re}\left( t^{\mathrm{TE}} \right)-\mathrm{Re}\left( r^{\mathrm{TE}} \right)\mathrm{Im}\left( t^{\mathrm{TE}} \right)}{\left( \mathrm{Re}\left( t^{\mathrm{TE}} \right) \right)^{2}+\left( \mathrm{Im}\left( t^{\mathrm{TE}} \right) \right)^{2}} \right)}{\sin\left( \frac{\mathrm{Im}\left( t^{\mathrm{TE}} \right)}{\left( \mathrm{Re}\left( t^{\mathrm{TE}} \right) \right)^{2}+\left( \mathrm{Im}\left( t^{\mathrm{TE}} \right) \right)^{2}} \right)}$ and $p_{2}=\frac{\arccos\left( \frac{\mathrm{Re}(t^{\mathrm{TE}})}{\left( \mathrm{Re}(t^{\mathrm{TE}}) \right)^{2}+\left( \mathrm{Im}(t^{\mathrm{TE}}) \right)^{2}} \right)+2m\pi}{k_{0}D}$,$m=0, \pm1, \pm2,\ldots$

Similarly, the effective parameters for TM polarization (magnetic field in the $y$ direction) can be obtained as:

$\varepsilon_{x,\mathrm{eff}}=\frac{q_{1}q_{2}}{\cos\theta_{i}}\pm\sqrt{\frac{({(q_{1})}^{2}+1){(q_{2})}^{2}}{\cos^{2} \theta_{i}}}$ (S13a)

$\mu_{y,\mathrm{eff}}-\frac{\sin^{2} \theta_{i}}{\varepsilon_{z,\mathrm{eff}}}-\varepsilon_{x,\mathrm{eff}}\cos^{2} \theta_{i}+2q_{1}q_{2}\cos\theta_{i}=0$ (S13b)

where $q_{1}=\frac{\left( \frac{\mathrm{Im}\left( r^{\mathrm{TM}} \right)\mathrm{Re}\left( t^{\mathrm{TM}} \right)-\mathrm{Re}\left( r^{\mathrm{TM}} \right)\mathrm{Im}\left( t^{\mathrm{TM}} \right)}{\left( \mathrm{Re}\left( t^{\mathrm{TM}} \right) \right)^{2}+\left( \mathrm{Im}\left( t^{\mathrm{TM}} \right) \right)^{2}} \right)}{\sin\left( \frac{\mathrm{Im}\left( t^{\mathrm{TM}} \right)}{\left( \mathrm{Re}\left( t^{\mathrm{TM}} \right) \right)^{2}+\left( \mathrm{Im}\left( t^{\mathrm{TM}} \right) \right)^{2}} \right)}$ and $q_{2}=\frac{\arccos\left( \frac{\mathrm{Re}(t^{\mathrm{TM}})}{\left( \mathrm{Re}(t^{\mathrm{TM}}) \right)^{2}+\left( \mathrm{Im}(t^{\mathrm{TM}}) \right)^{2}} \right)+2m\pi}{k_{0}d}$ , $m=0, \pm1, \pm2, \ldots$.Here,

$r^{\mathrm{TM}} =\frac{\left( \eta_{0}^{\mathrm{TM}}M_{22}^{\mathrm{TM}}-\eta_{0}^{\mathrm{TM}}M_{11}^{\mathrm{TM}} \right)-\left( \left( \eta_{0}^{\mathrm{TM}} \right)^{2}M_{12}^{\mathrm{TM}}-M_{21}^{\mathrm{TM}} \right)}{\left( \eta_{0}^{\mathrm{TM}}M_{22}^{\mathrm{TM}}+\eta_{0}^{\mathrm{TM}}M_{11}^{\mathrm{TM}} \right)-\left( \left( \eta_{0}^{\mathrm{TM}} \right)^{2}M_{12}^{\mathrm{TM}}+M_{21}^{\mathrm{TM}} \right)}$ (S14a)

$t^{\mathrm{TM}}=\frac{2\eta_{0}^{\mathrm{TM}}}{\left( \eta_{0}^{\mathrm{TM}}M_{22}^{\mathrm{TM}}+\eta_{0}^{\mathrm{TM}}M_{11}^{\mathrm{TM}} \right)-\left( \left( \eta_{0}^{\mathrm{TM}} \right)^{2}M_{12}^{\mathrm{TM}}+M_{21}^{\mathrm{TM}} \right)}$ (S14b)

where $\eta_{0}^{\mathrm{TM}}=1/cos \theta_{i}$, $M^{\mathrm{TM}} =M_{A}^{\mathrm{TM}}M_{B}^{\mathrm{TM}}M_{A}^{\mathrm{TM}}=\left( \begin{matrix} M_{11}^{\mathrm{TM}} & M_{12}^{\mathrm{TM}} \\ M_{21}^{\mathrm{TM}} & M_{22}^{\mathrm{TM}} \end{matrix} \right)$, $M_{A,B}^{\mathrm{TM}}=\left( \begin{matrix} \cos y_{A,B} & \left( iu_{A,B}/v_{A,B} \right)\sin y_{A,B} \\ \left( iv_{A,B}/u_{A,B} \right)\sin y_{A,B} & \cos y_{A,B} \end{matrix} \right)$ with $u_{A}^{2}=\mu_{A}-\frac{\sin^{2} \theta_{i}}{\varepsilon_{Az}}$, $v_{A}^{2}=\varepsilon_{Ax}$, $u_{B}^{2}=\mu_{B}-\frac{\sin^{2} \theta_{i}}{\varepsilon_{B}}$, $v_{B}^{2}=\varepsilon_{B}$, and $y_{A,B}=u_{A,B}v_{A,B}k_{0}d_{A,B}$.

From Eqs. (S12)-(S14), we can find out some properties of the effective parameters. 1) The effective parameters are non-unique, not only because of the multi-valued $m$ but also because there are only two independent equations to determine three unknown effective parameters for each polarization. Here, we emphasize that all solutions would lead to the same reflection, transmission coefficients as well as impedances. 2) The effective parameters are generally nonlocal as they rely on the incident angle $\theta_{i}$.

Next, we examine the validity of the effective parameters from the TO approach, i.e., $\varepsilon_{y}=\mu_{x}=\frac{1}{\mu_{z}}=\mu_{y}=\varepsilon_{x}=\frac{1}{\varepsilon_{z}}\approx0.71$. We find that these parameters indeed satisfy Eqs. (S12)-(S14) well. For further numerical verification, in Fig. S4, we present the field distributions in the MM (left) and the corresponding effective medium (right), showing almost identical field distributions in the air regions, for both TE (upper) and TM (lower) polarizations at different incident angles. These results further confirm that the designed MM indeed operates as an effective uniform medium with $\varepsilon_{y}=\mu_{x}=\frac{1}{\mu_{z}}=\mu_{y}=\varepsilon_{x}=\frac{1}{\varepsilon_{z}}\approx0.71$.


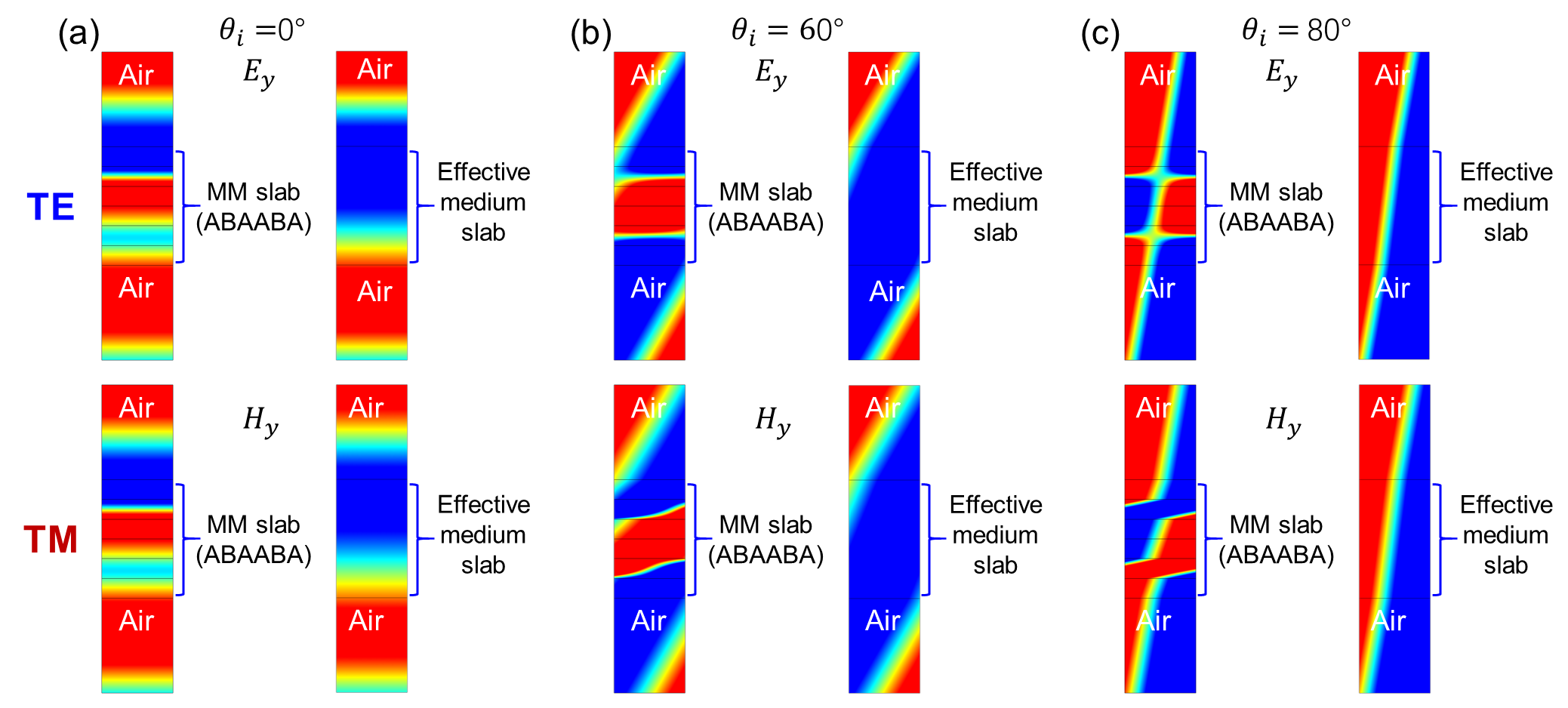


**Fig. S4.** Simulation distributions of electric fields (TE polarization, upper) and magnetic fields (TM polarization, lower) in the MM slab consisting of 2 ABA units (left) and the corresponding effective medium slab (right) at the incident angles of (a) 0$^{\circ}$, (b) 60$^{\circ}$, and (c) 80$^{\circ}$. The MM is adopted from Fig. 2 in the main text.

## Effective anisotropy and equivalence of “stretched free space”

The proposed pure-dielectric MMs are effectively anisotropic, and equivalent to “stretched free space”. Here, the effective anisotropy of the MM is defined as the ratio of semi-major axis (in the $k_{x}$ and $k_{y}$ directions) to semi-minor axis (in the $k_{z}$ direction) of the EFS ellipsoids. From the data in Fig. 2(e) in the main text, we find that the effective anisotropy is around 1.4. In the following, we perform numerical simulations to demonstrate the strong effective anisotropy and the equivalence of stretched free space, as well as the full-polarization omnidirectional impedance matching within the stretched free space.

According to transformation optics (TO), when stretching a layer of free space along the $z$ direction via the coordinate transformation $x'=x$, $y'=y$, and $z'=\kappa z$, the stretched free space is characterized by a relative permittivity of $\varepsilon=\left( \begin{matrix} \varepsilon_{x} & & \\ & \varepsilon_{y} & \\ & & \varepsilon_{z} \end{matrix} \right)=\left( \begin{matrix} 1/\kappa& & \\ & 1/\kappa& \\ & & \kappa\end{matrix} \right)$ and a relative permeability of $\mu=\left( \begin{matrix} \mu_{x} & & \\ & \mu_{y} & \\ & & \mu_{z} \end{matrix} \right)=\left( \begin{matrix} 1/\kappa& & \\ & 1/\kappa& \\ & & \kappa\end{matrix} \right)$, where the original free space resides in space $\{x,y,z\}$, the stretched free space occupies the space $\{x',y',z'\}$, and $\kappa$ is the stretching ratio^8^. We that $\varepsilon=\mu$ for the stretched free space, indicating the polarization-independent property. In the following, we take the transverse-electric (TE) polarization (electric field along the $y$ direction) as an example to demonstrate the full-polarization omnidirectional impedance matching within the stretched free space.

We assume a TE polarized wave with a wave vector of $\mathbf{k}=k_{0}\sin\theta\hat{x}+k_{0}\cos\theta\hat{z}$ incident on the interface between free space and the stretched free space (on $xy$ plane). Here, $\theta$ is the incident angle. The reflection coefficient at this interface can be obtained based on the boundary continuity conditions as^1^:

$r^{\mathrm{TE}}=\frac{\mu_{x}k_{0}\cos\theta-k_{z}}{\mu_{x}k_{0}\cos\theta+k_{z}}$ (S15)

where $k_{z}$ is the $z$-component of wave vector in the stretched free space. According to the dispersion of the stretched free space, that is, $\frac{k_{x}^{2}}{\mu_{z}}+\frac{k_{z}^{2}}{\mu_{x}}=\varepsilon_{y}k_{0}^{2}$, we have $k_{z}=\sqrt{\mu_{x}\varepsilon_{y}k_{0}^{2}-\frac{\mu_{x}}{\mu_{z}}k_{0}^{2}\sin^{2} \theta}$. Then, Eq. (S15) can be re-formulated as,

$r^{\mathrm{TE}}=\frac{\cos\theta-\sqrt{\frac{\varepsilon_{y}}{\mu_{x}}-\frac{1}{\mu_{x}\mu_{z}}\sin^{2} \theta}}{\cos\theta+\sqrt{\frac{\varepsilon_{y}}{\mu_{x}}-\frac{1}{\mu_{x}\mu_{z}}\sin^{2} \theta}}$ (S16)

Since $\mu_{x}=\varepsilon_{y}=1/\kappa$ and $\mu_{z}=\kappa$, we see from Eq. (S16) that $r^{\mathrm{TE}}=0$, independent of the incident angle $\theta$. This indicates that the stretched free space is omnidirectional impedance matched with free space. Considering the polarization-independent property within this stretched free space, we would obtain the same conclusion for transverse-magnetic polarization. Therefore, the stretched free space exhibits full-polarization omnidirectional impedance matching.

In section 5, we find that the effective parameters of the proposed MM consisting of even-numbered units are:

$\varepsilon_{x,eff}\approx\varepsilon_{y,eff}\approx\mu_{x,eff}\approx\mu_{y,eff}\approx0.71$, $\frac{1}{\varepsilon_{z,eff}}\approx\frac{1}{\mu_{z,eff}}\approx\frac{1}{0.71}$ (S17)

Equation (S17) indicates the whole MM exhibits the effective magnetic response, although it is composed of nonmagnetic dielectrics. Moreover, the effective parameters approximately satisfy the requirement in parameters of a stretched free space. Therefore, we see that although the dielectric components of the MM are anisotropic only in permittivity, the whole MM exhibits anisotropic effective permittivity and permeability in the same way as those in TO-based stretched free space.


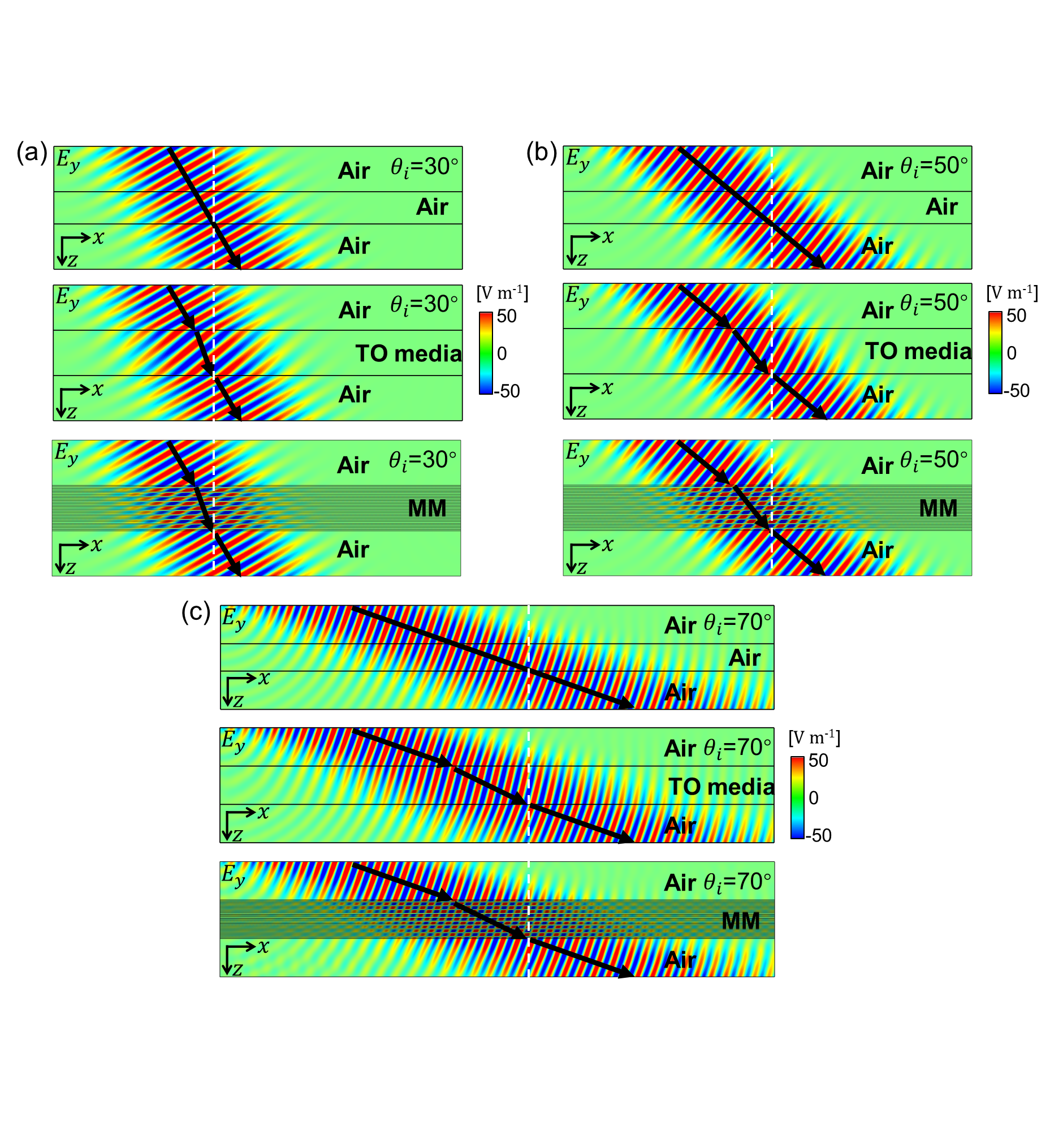


**Fig. S5.** Distributions of $E_{y}$ when a TE-polarized Gaussian beam is incident onto a virtual slab of air (thickness 7.1$a$, upper), a TO medium slab (thickness 10$a$, middle) and a MM slab (10 unit cells, lower) under the incident angles of (a) $30^{\circ}$, (b) $50^{\circ}$, (c) $70^{\circ}$.

Then, we perform numerical simulations for further verification. Here, we assume that the dispersion of the TO medium is the same as that of our proposed MM at the working frequency ${fa}/c=0.277$, except for the shift in the $k_{z}$ direction. Considering the fitted dispersion of the MM, we find out the parameters of the TO medium as $\varepsilon_{y}=\mu_{x}=\frac{1}{\mu_{z}}=0.71$ for the TE polarization, and $\mu_{y}=\varepsilon_{x}=\frac{1}{\varepsilon_{z}}=0.71$ for the TM polarization. According to the theory of TO, the thickness of the TO medium is found to be $1.4d_{0}$.

The wave behaviors on a layer of free space (thickness $d_{0}$), a TO medium slab (thickness $1.4d_{0}$) and the MM slab (thickness $1.4d_{0}$) shall be the same, irrespective of the incident angles and polarization states, except that a phase difference between the transmitted waves may occur due to the shift of dispersion of the MM in the $k_{z}$ direction. For verification, we examine the wave reflection and refraction on a virtual slab of air (thickness 7.1$a$), a TO medium slab (thickness 10$a$) and a MM slab (10 units) under the illumination of a Gaussian beam. Figures S5(a)-S5(c) show the simulated field distributions for the TE polarization under the incident angles of $30^{\circ}$, $50^{\circ}$ and $70^{\circ}$, respectively. The upper, middle and lower panel graphs correspond to the virtual slab of air, the TO medium slab and the MM slab, respectively. We see that the wave behaviors in the three cases are almost the same, including the zero-reflection property, the phases and positions of transmitted waves, irrespective of the incident angles. Similar wave behaviors are also observed for the TM polarization (Fig. S6).


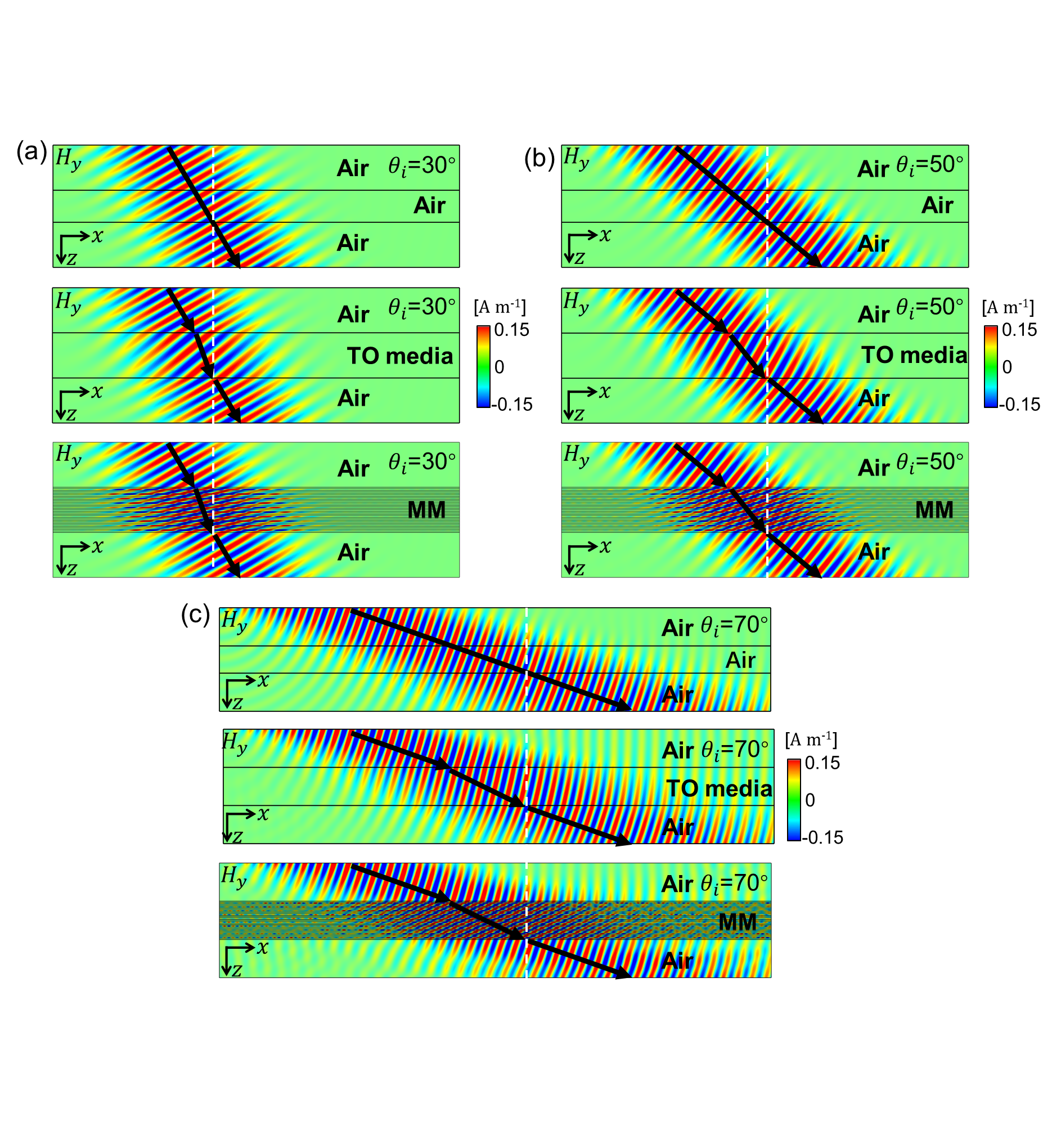


**Fig. S6.** Distributions of $H_{y}$ when a TM-polarized Gaussian beam is incident onto a virtual slab of air (thickness 7.1$a$, upper), a TO medium slab (thickness 10$a$, middle) and a MM slab (10 units, lower) under the incident angles of (a) $30^{\circ}$, (b) $50^{\circ}$, (c) $70^{\circ}$.

The above simulation results have two implications. The first is that the MM slab is indeed equivalent to “stretched free space”. The second is that the optical anisotropy of the TO medium and the MM is effectively the same. We know that the anisotropy of the TO medium can be well defined as $\frac{\sqrt{\varepsilon_{y}\mu_{z}}}{\sqrt{\varepsilon_{y}\mu_{x}}}=1.4$ (or $\frac{\sqrt{\mu_{y}\varepsilon_{z}}}{\sqrt{\mu_{y}\varepsilon_{x}}}=1.4$) for the TE (or TM) polarization. Therefore, the MM is expected to possess a strong effective anisotropy as 1.4.

## Experimental setup, measurement methods, and supplemental simulation results

The experiment was performed in an anechoic chamber with a Keysight PNA-X 5242A Network Analyzer. Figure S7 shows the picture of experimental setup. A dipole antenna is used as the signal source to generate electromagnetic waves, and another one is used to probe the near-field electric fields. The input signal has a power of 0 dBm and passes through a 23 dBm amplifier. Both the dipole antennas are placed horizontally along $y$ direction, therefore only the $E_{y}$-distributions are detected here. The source antenna is placed at a distance of 39.5 mm from the center of the fabricated MM sample. The detecting probe is mounted to a computer-controlled translational stage and scan at a precision of 3 mm per step. The scanning areas are located on both the $xz$ and $yz$ planes before and after the MM sample. The scanning areas are 300$\times$240 mm^2^ each, which are marked by dashed lines in Fig. 4b in the main text. Each scanning area has a distance of 30 mm from the source antenna.


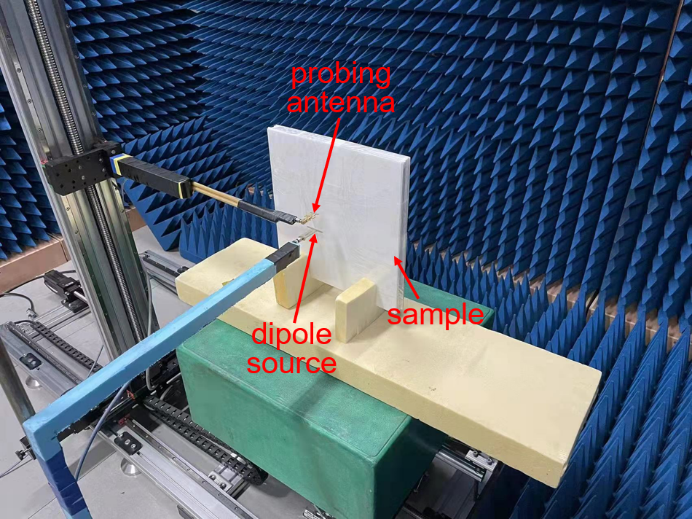


**Fig. S7.** Picture of experimental setup for the measurement of near-field electric fields.

In the main text, the experimentally measured $y$-component of electric field $E_{y}^{\mathrm{MM}}$ is presented in Fig. 4. Here, we’d like to show the corresponding simulation results for comparison. Figure S8(a) shows a three-dimensional view of the simulated distribution of $E_{y}^{\mathrm{MM}}$. The left panels in Figs. S8(b) and S8(c) show, respectively, $E_{y}^{\mathrm{MM}}$-distributions on $xz$ and $yz$ planes. A well-defined cylindrical wave pattern is observed on $xz$ plane, corresponding to the quasi-TE polarization. And a dipole radiation pattern is observed on $yz$ plane, corresponding to the quasi-TM polarization. On both the $xz$ and $yz$ planes, the scattering fields (i.e. $E_{y}^{\mathrm{MM}}-E_{y}^{\mathrm{Air}}$ with $E_{y}^{\mathrm{Air}}$ being the electric field in the absence of the MM sample) are very weak, as presented in the right panels in Figs. S8(b) and S8(c). These results match well with the experimental observation in Fig. 4, and further confirm the full-polarization and omnidirectional generalized Brewster effect in the dielectric MM.


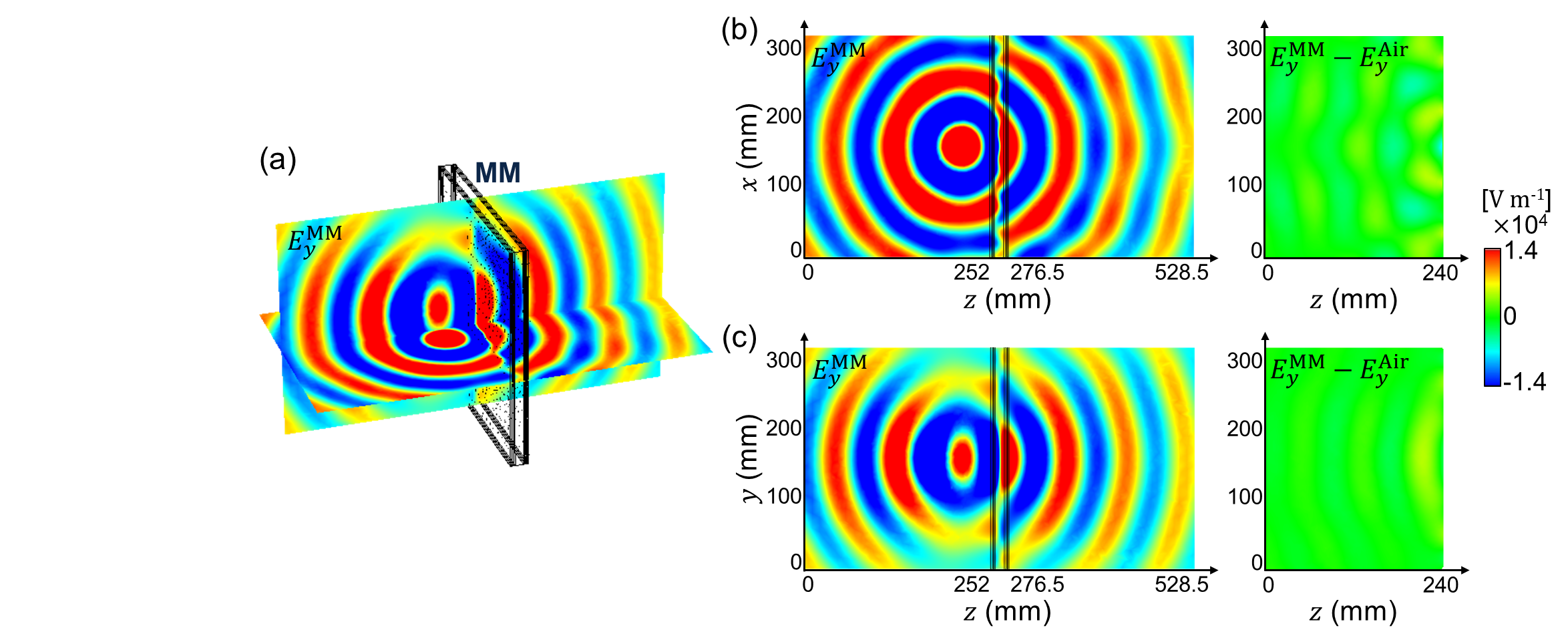


**Fig. S8.** (a) A three-dimensional view of simulated $E_{y}^{\mathrm{MM}}$-distribution when a dipole source polarized along the $y$ direction is placed nearby the MM sample at a distance of 39.5 mm from the center of the sample. (b) The $E_{y}^{\mathrm{MM}}$-distribution on $xz$ plane (left) and the scattering field $E_{y}^{\mathrm{MM}}{-E}_{y}^{\mathrm{Air}}$ on the source side (right). (c) The $E_{y}^{\mathrm{MM}}$-distribution on $yz$ plane (left) and the scattering field $E_{y}^{\mathrm{MM}}{-E}_{y}^{\mathrm{Air}}$ on the source side (right).


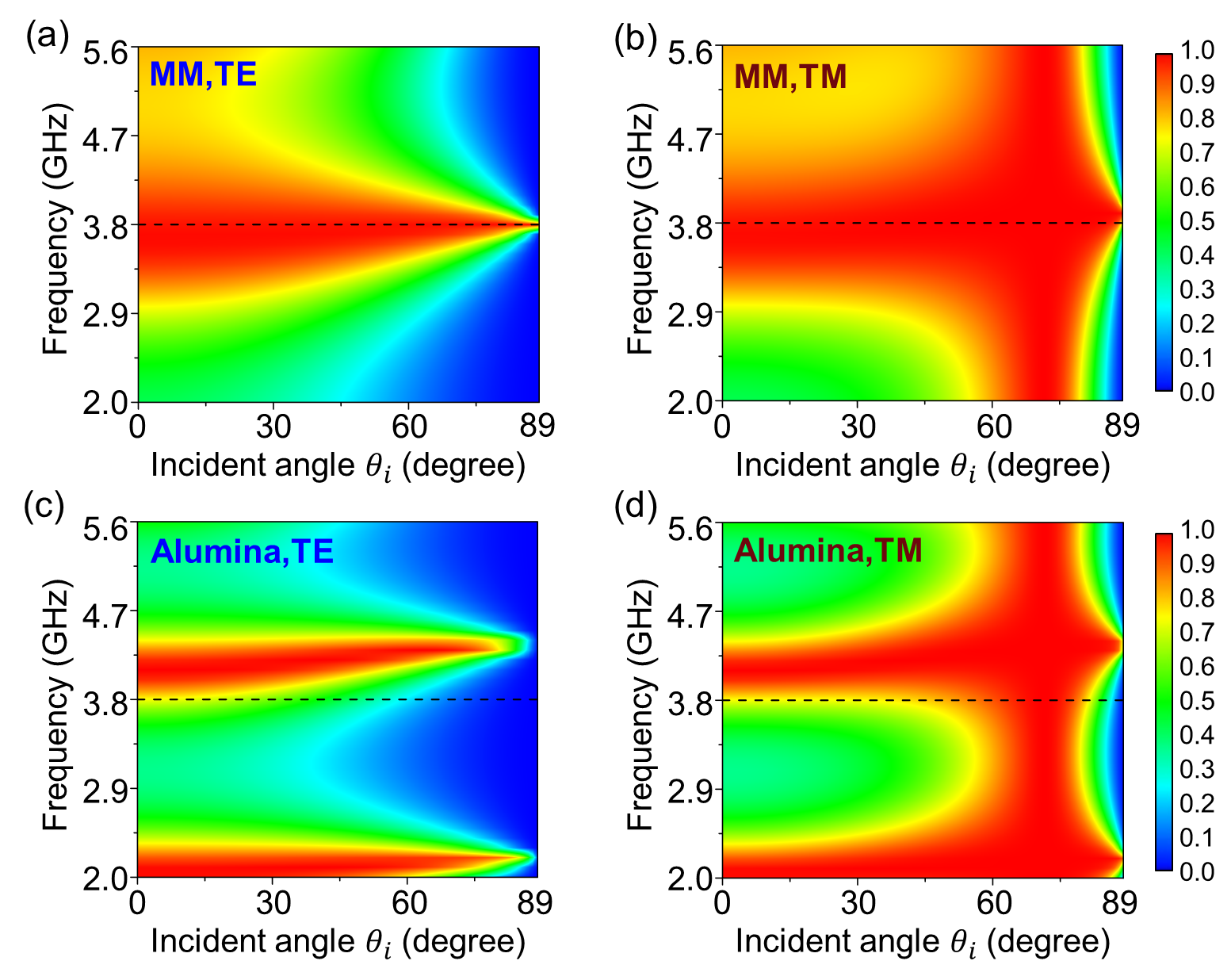


**Fig. S9.** Simulated transmittance through the MM sample slab (one unit cell) as functions of the incident angle and working frequency for the (a) TE, (b) TM polarization.

In addition, we’d like to show the bandwidth of high transmission of the experimental MM sample. Figures S9(a) and S9(b) show the simulated transmittance through the MM sample slab (one unit cell) as functions of the incident angle and working frequency for TE and TM polarizations, respectively. Near-perfect transmission for both polarizations is observed at 3.8 GHz. We find that the bandwidth of high transmission (>0.9) is considerably large under small incident angles. As the increase of the incident angle, the bandwidth decreases for the TE polarization, while can be even larger and becomes ultra-broad near the predetermined Brewster’s angle (i.e. 71.5°) for the TM polarization.


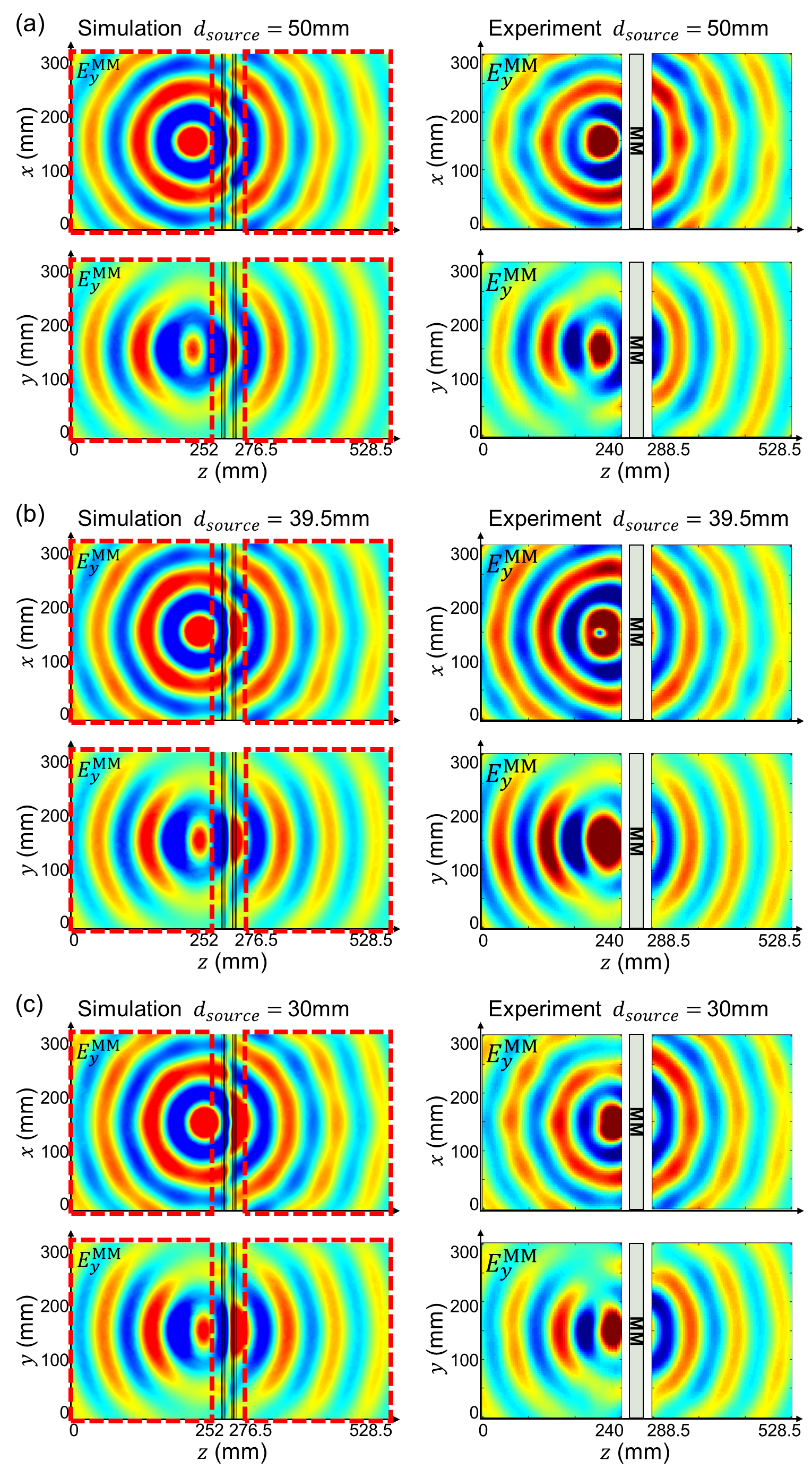


**Fig. S10.** Simulated (left) and measured (right) $E_{y}^{\mathrm{MM}}$-distributions on $xz$ (upper) and $yz$ (lower) planes at 3.8 GHz when a dipole source polarized along the $y$ direction is placed nearby the metamaterial sample at the distance of (a) 50 mm, (b) 39.5 mm, (c) 30 mm from the center of the sample.

Furthermore, to verify our sample is not some kind of resonant tunneling effect for a specific sample thickness (of the order of $\lambda_{0}/2$) and for a specific distance between the source and the sample (of the same order), we have carried out simulations and the corresponding experiments by varying the distance between the dipole source (polarized along the $y$ direction) and the sample. The results are plotted in Figure S10, the experimental scanning areas (marked with red dashed lines in simulations) are 300$\times$240 mm^2^ in dimension and are located on $xz$ and $yz$ planes on the both sides of the metamaterial sample. Each scanning area has a distance of 30 mm from the source antenna. All the results of simulations and the corresponding experiments are consistent with each other, confirming the low reflectance property independent of the distance between the source and sample.

## More examples of MM radomes


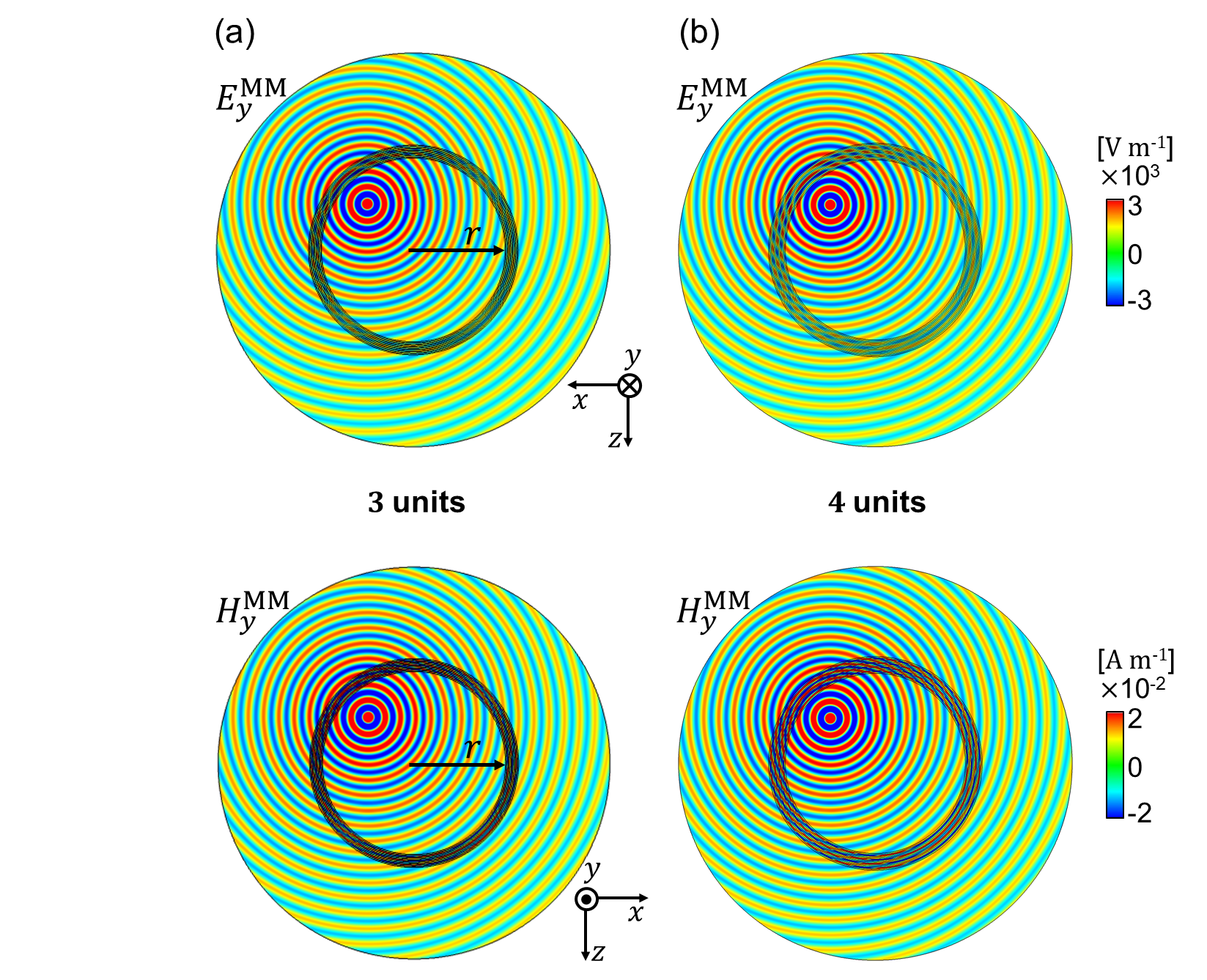


**Fig. S11.** [(a) and (b)] Simulated distributions of electric fields radiated from an out-of-plane electric monopolar source, placed off-center within a circular MM radome composed of (a) 3, (b) 4 ABA units.

[(c) and (d)] Simulated distributions of magnetic fields radiated from an out-of-plane magnetic monopolar source, placed off-center within a circular MM radome composed of (c) 3, (d) 4 ABA units. The MM model is adopted from Fig. 2.

In this section, we demonstrate two examples of MM radomes comprising 3 (or 4) ABA units, as shown in Figs. S11(a) and S11(c) [or Figs. S11(b) and S11(d)]. Similar to the example in Fig. 5 in the main text. Near-total transmission, independent of the number of the units, is observed. These results further underscore the validity of using the self-dual MM to construct radomes that are totally wave-transparent to electromagnetic signals, without causing any distortion in wavefronts or attenuation in amplitudes. These results also show that the MM radomes can have a flexible thickness.

## A practical realization of microwave self-dual metamaterial

Besides the special solution described in Eq. (2) in the main text (i.e. the design in Fig. 4), there exist other ways to realize practical pure-dielectric MMs. Here, we demonstrate another example utilizing relatively high-index materials. The dielectric MM is composed of ABA multilayer with $d_{A}=3.15$mm, $d_{B}=3.7$mm and $\varepsilon_{B}=25$. As shown schematically in Fig. S12(a), each A layer consists of five units of periodically stacked dielectric C layer with drilling holes ($\varepsilon_{C}=28.2$, $d_{C}=32.76 \mu$m, $r=157.5 \mu$m) and free-space layer ($d_{D}=597.24 \mu$m). The holes on the C layers are arranged in a square lattice with lattice constant 0.63 mm. Based on quasi-static effective medium theory, the effective parameters of the composite A layer are $\varepsilon_{Ax,\mathrm{eff}}=\varepsilon_{Ay,\mathrm{eff}}\approx1.95$ and $\varepsilon_{Az,\mathrm{eff}}\approx1.05$, approximately satisfy the condition in Eq. (1). The functioning frequency is 7.67 GHz. Figs. S12(b) and S12(d) show, respectively, the transmittance through the dielectric MM slab with $N$ (=1, 2, 3) number of unit cells for TE and TM polarizations at 7.67 GHz. Near-perfect transmission is achieved under almost all incident angles for both polarizations, irrespective of the number of units $N$. For comparison, we also plot the transmittance through the dielectric B layer alone with different thicknesses, as shown by solid lines in Figs. S12(b) and S12(d). Clearly, the transmission is much lower and varies with the thickness, except at the traditional Brewster’s angle of 78.7° for the TM polarization. From the transmittance as a function of frequency under different incident angles for $N=1$, as shown in Figs. S12(c) and S12(e) for TE and TM polarizations, respectively, we find that the bandwidth of high transmission (>0.9) is considerably large (~0.15 GHz) even at 80°. We note that such a bandwidth is sufficiently large for wireless communication. For the TM polarization, the bandwidth is even larger and becomes ultra-broad near the traditional Brewster’s angle, i.e., 78.7$^{\circ}$.


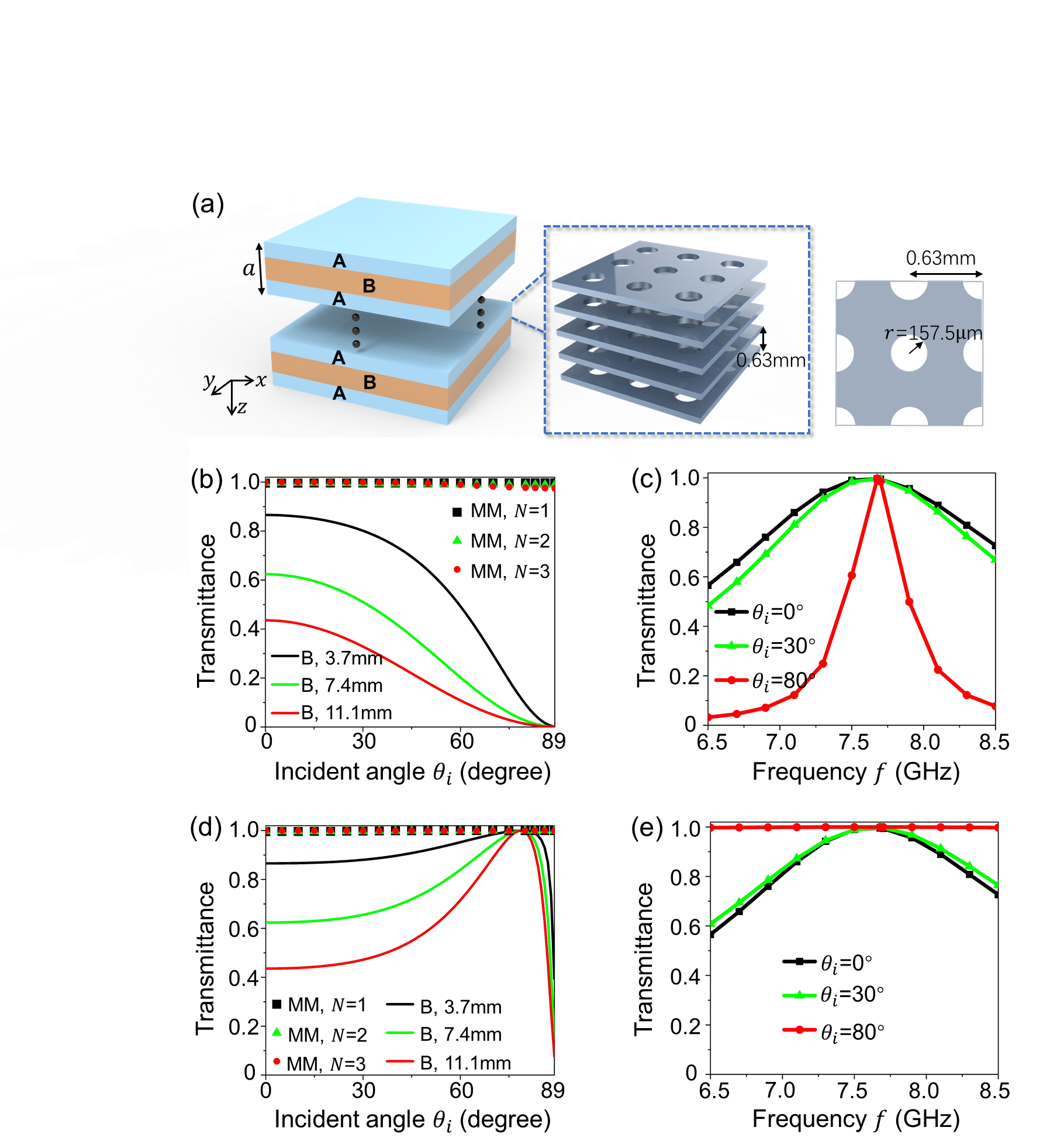


**Fig. S12.** (a) Schematic layout of a design of pure-dielectric MM. The right panel graphs show the realization of the anisotropic layer A by periodically stacking isotropic dielectric slabs with drilling holes. [(b) and (d)] Transmittance with respect to the incident angle for (b) TE-, (d) TM-polarized waves incident on the MM slab with $N$ number of units (dots), and the dielectric B layer with different thicknesses (solid lines) at 7.67 GHz. [(c) and (e)] Transmittance through the MM slab ($N=1$) as a function of the functioning frequency under different incident angles for (c) TE and (e) TM polarizations.

## More examples of self-dual pure-dielectric metamaterials

The proposed approach to design pure-dielectric MMs exhibiting the artificial duality symmetry and full-polarization omnidirectional Brewster effect is general, and is quite flexible in the selection of dielectric materials. And we also discussed the corresponding bandwidth (the transmittance$T>0.9$) at the incident angles $\theta_{i}=0^{\circ}$and $\theta_{i}=80^{\circ}$. Here, we show more examples, as summarized in Table S1. It is noted that condition of $\log\left| \eta\right|<-1$ indicates that the reflected energy at the air-MM interface is less than 1%. We find that for any dielectric B with $\varepsilon_{B}\geq3$, we can find out appropriate anisotropic dielectric A and optimized thicknesses to realize the near-perfect Brewster effect for a chosen working frequency, thus providing the potential of extension to higher frequencies including the infrared and optical regimes.

**Table S1.** More examples of pure-dielectric MMs exhibiting the artificial duality symmetry, full-polarization omnidirectional Brewster effect and bandwidth

| $\varepsilon_{B}$ | $\varepsilon_{Ax}\boldsymbol{=}\varepsilon_{Ay}$ | $\varepsilon_{Az}$ | $d_{B}\boldsymbol{/}a$ | $fa\boldsymbol{/}c$ | $\log\left\vert\eta\right\vert\boldsymbol{<-}1$ | Bandwidth  @$\theta_{i}\mathbf{=}0\boldsymbol{^{\circ}}$ | Bandwidth  @$\theta_{i}\mathbf{=}80\boldsymbol{^{\circ}}$ |
| --- | --- | --- | --- | --- | --- | --- | --- |
| 3 | 1.2 | 3/2.8 | 0.37 | 0.799 | TE: $0\leq\theta_{i}\leq85^{\circ}$  TM: $0\leq\theta_{i}\leq82^{\circ}$ | 0.649 | 0.083 |
| 8 | 1.2 | 8/7.8 | 0.37 | 0.485 | TE: $0\leq\theta_{i}\leq86^{\circ}$  TM: $0\leq\theta_{i}\leq86^{\circ}$ | 0.207 | 0.033 |
| 15 | 1.2 | 15/14.8 | 0.37 | 0.352 | TE: $0\leq\theta_{i}\leq88^{\circ}$  TM: $0\leq\theta_{i}\leq86^{\circ}$ | 0.131 | 0.022 |
| 25 | 1.2 | 25/24.8 | 0.37 | 0.272 | TE: $0\leq\theta_{i}\leq82^{\circ}$  TM: $0\leq\theta_{i}\leq89^{\circ}$ | 0.094 | 0.016 |
| 25 | 2 | 25/24 | 0.37 | 0.256 | TE: $0\leq\theta_{i}\leq85^{\circ}$  TM: $0\leq\theta_{i}\leq84^{\circ}$ | 0.117 | 0.019 |
| 25 | 2 | 25/24 | 0.3375 | 0.277 | TE: $0\leq\theta_{i}\leq89^{\circ}$  TM: $0\leq\theta_{i}\leq83^{\circ}$ | 0.128 | 0.020 |
| 45 | 1.5 | 45/44.5 | 0.37 | 0.200 | TE: $0\leq\theta_{i}\leq81^{\circ}$  TM: $0\leq\theta_{i}\leq88^{\circ}$ | 0.070 | 0.012 |

## A practical realization of infrared self-dual metamaterial based on silicon

In this section, we demonstrate an infrared self-dual MM made of silicon (relative permittivity ~11.7). Similar to the experimental example in Fig. 4 in the main text, we first use subwavelength silicon-air multilayers to realize effective anisotropic A layers, and then construct the ABA unit where B is also made of silicon. The designed ABA unit is schematically shown in Fig. S13(a). The thickness of the side thin silicon layers is $64$ nm, which are spaced by $624$ nm-thickness air gaps. In this case, the effective A layer has a thickness of 2064 nm, and possess effective parameters of $\varepsilon_{Ax}\approx2.00$ and $\varepsilon_{Az}\approx1.09$, approximately satisfying the condition in Eq. (1) in the main text. The thickness of central thick silicon layer is optimized as 2.8 $\mu$m. Such a silicon-based MM exhibits nearly full-polarization omnidirectional Brewster effect, as demonstrated by the high transmission for nearly all incident angles and both polarizations [Fig. S13(b)].


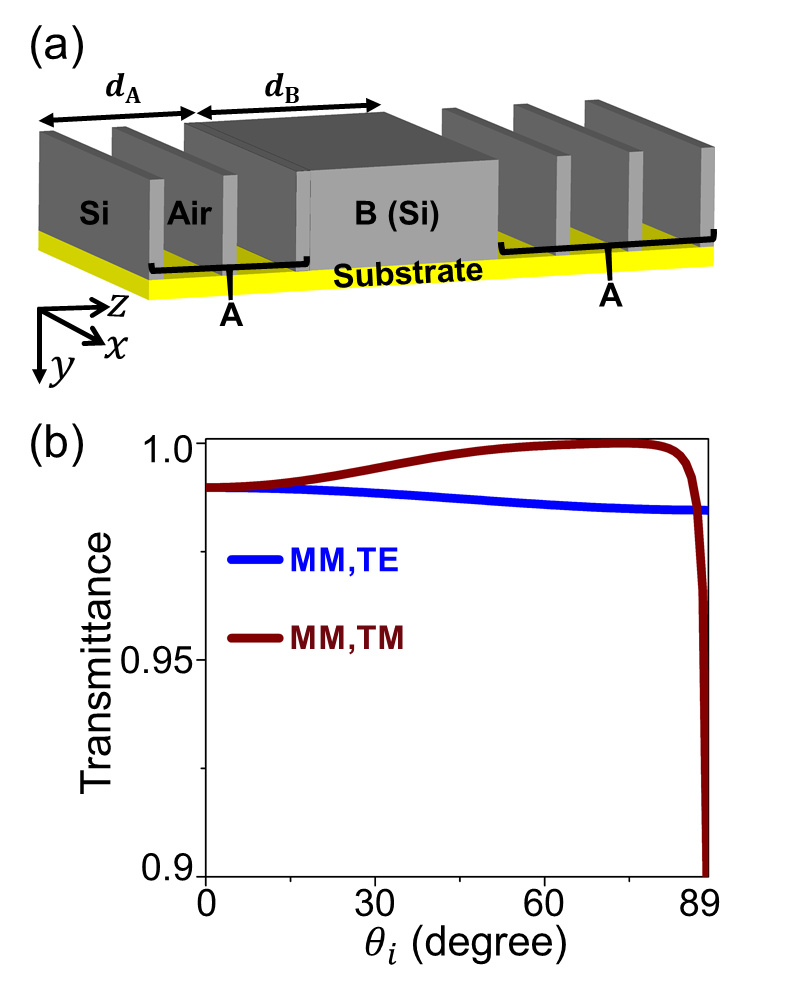


**Fig. S13.** (a) Illustration of the unit cell of the designed infrared MM consisting of silicon layers spaced by air. (b) Simulated transmittance through the MM sample for TE (blue) and TM (red) polarizations as a function of the incident angle at 14.141 THz.

**References**

1. Huang, H. & Shen, Z. Angle-selective surface based on uniaxial dielectric-magnetic slab. *IEEE Antennas Wirel. Propag. Lett.* **19**, 2457-2461 (2020).

2. Luo, J. *et al.* Ultratransparent media and transformation optics with shifted spatial dispersions. Physical Review Letters*.* **117**, 223901 (2016).

3. Guo, J. *et al.* Electromagnetically large cylinders with duality symmetry by hybrid neural networks. Optics & Laser Technology **168**, 109935 (2024).

4. Smith, D. R. & Schultz, S. Determination of effective permittivity and permeability of metamaterials from reflection and transmission coefficients. Physical Review B **65**, 195104 (2002).

5. Berreman, D. W. Optics in stratified and anisotropic media: 4×4-matrix formulation. Journal of the Optical Society of America 62, 502-510 (1972).

6. Huang, M., Li, X. & Luo, J. All-dielectric unidirectional complementary media for transmission enhancement. Optics Express 28, 33263-33273 (2020).

7. Macleod, H. A., *Thin-Film Optical Filters*, 4th ed. (CRC Press, 2010).

8. He, Q. et al. Optic-null medium: realization and applications. Optics Express 21, 28948-28959 (2013).
